# Short-term effects of combined environmental factors on respiratory disease mortality in Qingdao city: A time-series investigation

**DOI:** 10.1371/journal.pone.0318250

**Published:** 2025-01-28

**Authors:** Xin Zhang, Zijian Xi, Min Yang, Xiuqin Zhang, Ruikai Wu, Shuang Li, Lu Pan, Yuan Fang, Peng Lv, Yan Ma, Haiping Duan, Bingling Wang, Kunzheng Lv

**Affiliations:** 1 Department of Science and Technology, Yancheng First People’s Hospital, Yancheng, Jiangsu, China; 2 School of Public Health, Binzhou Medical University, Yantai, Shandong, China; 3 Shibei District Center for Disease Control and Prevention, Qingdao, Shandong, China; 4 Department of Environmental Health, Qingdao Municipal Center for Disease Control and Prevention, Qingdao Institute of Disease Prevention, Qingdao, Shandong, China; 5 School of Public Health, Xinjiang Medical University, Urumqi, Xinjiang, China; 6 Qingdao Eco-Environmental Monitoring Center of Shandong Province, Qingdao, Shandong, China; 7 Qingdao Meteorological Bureau (Qingdao Marine Meteorological Bureau), Qingdao, Shandong, China; Kyung Hee University School of Medicine, REPUBLIC OF KOREA

## Abstract

**Background:**

It is crucial to comprehend the interplay between air pollution and meteorological conditions in relation to population health within the framework of "dual-carbon" targets. The purpose of this study was to investigate the impact of intricate environmental factors, encompassing both meteorological conditions and atmospheric pollutants, on respiratory disease (RD) mortality in Qingdao, a representative coastal city in China.

**Methods:**

The RD mortality cases were collected from the Chronic Disease Surveillance Monitoring System in Qingdao during Jan 1st, 2014 and Dec 31st, 2020. The distributed-lag nonlinear model and generalized additivity model were used to assess the association between daily mean temperature (DMT), air pollutant exposure and RD mortality. To ascertain the robustness of the model and further investigate this relationship, a stratified analysis and sensitivity analysis were conducted to mitigate potential confounding factors.

**Results:**

A total of 19,905 mortalities from RD were recorded. The minimum mortality temperature (MMT) was determined to be 23.5°C, and DMT and RD mortality showed an N-shaped relationship. At the MMT of 23.5°C, the cumulative relative risk (cumRR) for mortality within a lag period of 0–14 days from the highest temperature (31°C) was estimated at 2.114 (95% confidence interval [CI]: 1.475 ~ 3.028). The effect value of particulate matter (PM) also increased with a longer cumulative lag time. In the single pollutant model, the highest risk of RD mortality was observed on the lag1-day of per 10 μg/m^3^ increase in PM_2.5_ exposure, with an excess risk ratio (ER) of 0.847% (95% CI: 0.335% ~ 1.362%). The largest cumulative effect was found at a lag of 8 days, with an ER of 1.546% (95% CI: 0.483% ~ 2.621%). A similar trend was found for PM_10_. For O_3_ exposure, the highest risk was observed on the lag1-day of per 10 μg/m^3^ increase, with an ER of 1.073% (95% CI: 0.502% ~ 1.647%), and the largest cumulative effect occurred at a lag of 2 days with an ER of 1.113% (95%CI: 0.386% ~ 1.844%). Results from the dual-pollutants model demonstrated that the effect of PM on the risk of RD mortality remained significant and slightly increased in magnitude. Moreover, composite pollutants exhibited a higher risk effect, reaching its peak after one week; however, there was a decrease in single-day cumulative effects as more pollutant types were included. Subgroup analysis showed that females, elderly individuals, and those exposed during warm seasons demonstrated greater susceptibility to PM exposure.

**Conclusion:**

The present study revealed a significant association between short-term exposure to high temperature, PM_2.5_, PM_10_ and O_3_ and the risk of RD mortality in Qingdao, even in dual- and composite-pollutants models. Furthermore, our findings indicate that females, the elderly population, and warm seasons exhibit heightened sensitivity to PM exposure.

## 1. Introduction

Air pollution and climate change have emerged as the foremost global health threats of the 21st century, occupying the top position on the WHO’s list of 10 leading health threats. According to the Global Burden of Disease (GBD) 2019 statistics, air pollution ranks as the fourth leading risk factors for mortality across 204 countries and territories, while uncomfortable temperatures rank eleventh [1]. In China, air pollution stands as the fourth leading mortality risk factor, with uncomfortable temperatures ranking eighth [1]. The study reveals that environmental factors contribute to a staggering 6.67 million global deaths, with air pollution alone accounting for a significant proportion of these deaths, at 53%. Additionally, approximately 1.95 million premature mortalities occur annually worldwide due to uncomfortable temperatures [1]. Chronic respiratory diseases (RD), which include conditions affecting the respiratory tract and lung structures [2], represent a major cause of morbidity and mortality across nations [3]. Globally in 2017, RD ranked third among all causes of mortality, contributing to approximately 7.0% (95% uncertainty interval [UI] 6.8 ~ 7.2%) [4]. A systematic review by Agarwal et al. in 2019 indicated that chronic RD account for a significant proportion of total deaths, with estimates suggesting they contribute to around 30% of all deaths in certain populations [5]. Understanding factors influencing the mortalities of RD has long been an area of concern.

Meteorological conditions can facilitate the accumulation of air pollution, and air pollutants can react with various meteorological parameters. These interactions have the potential to exacerbate respiratory illnesses by interacting with inhaled allergens. Rahman et al. [6] discovered that co-exposure to particulate air pollution and extreme heat significantly increased mortality risk compared to single exposure to either air pollution or extreme heat alone. Moreover, it was observed that both air pollution and meteorological conditions independently and synergistically impact respiratory health in Europe [7]. Due to variations in study areas and levels of exposure to air pollutants, heterogeneous health effects on populations were indicated [8]. A multi-center research has demonstrated that while air pollution is a global issue, local solutions are necessary [9]. With China’s commitment to peak carbon emissions by 2030 and achieve carbon neutrality by 2060, there will be profound implications for the country’s air quality, climate, and overall public health [8]. In the context of China’s "two-carbon" goals, understanding the interactions between air pollution and meteorological conditions becomes crucial for comprehending the relationship between environment and health. Simultaneously, changes in pollutant emissions and meteorological conditions under these goals are expected to have significant far-reaching consequences on public health.

Currently, both domestic and international studies on the impact of air pollution and meteorological changes on the mortality of RD primarily focus on infectious diseases, asthma, allergic rhinitis, lung cancer and chronic obstructive pulmonary disease [10]. However, studies specific to Qingdao mainly concentrate on pneumonia [11] and lung cancer [12], neglecting other RDs. Therefore, it is imperative to conduct comprehensive research and analysis considering combined environmental factors affecting the mortality of RD in Qingdao. Qingdao is situated in East China, southeast of the Shandong Peninsula and east of the Yellow Sea. Located within the north temperate monsoon region, Qingdao experiences a distinct maritime climate due to its direct interaction with the marine environment. Influenced by oceanic surface winds, sea currents, and water masses brought about by the southeast monsoon, its urban area exhibits notable characteristics associated with this climatic pattern, which hold significant implications for global research on the impact of climate change and complex environmental factors. Therefore, our study aimed to assess the short-term exposure risk of RD mortality outcomes in the population of Qingdao during 2014–2020 by integrating meteorological conditions and atmospheric pollutants. This assessment aims to provide a scientific foundation for conducting health risk assessments related to climate change and evaluating the adaptive capacity of the population in Qingdao. Additionally, it seeks to enhance monitoring and early warning systems for climate-sensitive diseases, particularly those affecting the respiratory system, while also addressing synergic effects between major pollutants and key climate changes. Ultimately, this research aims to contribute scientific evidence towards understanding how global environmental changes affect population health.

## 2. Materials and methods

### 2.1 Respiratory disease mortality data

The number of daily mortalities attributed to respiratory diseases (ICD-codes: J00 –J98), according to the International Classification of Diseases (ICD-10) for newly diagnosed conditions, was recorded. The case data covering the period from January 1, 2014 to December 31, 2020 was obtained in March 2023 from the Chronic Disease Surveillance Monitoring System in Qingdao. This was done after receiving approval from the Ethics Committee of Qingdao Municipal Center for Disease Control and Prevention. No personally identifiable information was collected during or after data collection. The necessity of informed consent was deemed unnecessary by the board.

### 2.2 Atmospheric pollutants and meteorological data

As of 2020, Qingdao administers seven municipal districts and three county-level cities. The city has seven national meteorological observation stations as well as twenty-five air quality monitoring stations. The data on atmospheric pollutants and meteorological conditions for the period 2014–2020 were provided in March 2023 by the Qingdao Eco-Environmental Monitoring Center of Shandong province and the Qingdao Meteorological Observatory, respectively. The Atmospheric pollutant data include the daily average concentrations (DAC) of PM_2.5_, PM_10_, SO_2_, NO_2_, CO_2_, and the maximum DAC of O_3_-8h. Each monitoring station and each pollutant should have at least 27 daily average concentration values per month (at least 25 in February) and a minimum of 324 daily average concentration values per year. To verify if there are any abnormal data points, the average value from each station distributed across districts/counties is taken as the pollutants’ concentrations for that day. Any value less than 0 is considered abnormal. If any abnormal values are found, they should be traced and verified before being correcting accordingly. Overall, the quality of the air pollutant data was good. The meteorological data consist of daily mean temperature (DMT), diurnal temperature range (DTR), relative humidity (RH), wind speed (WS), air pressure (AP), and precipitation (P). It is important to ensure that there are no missing or abnormal values in the daily meteorological data. The missing rate should be below 5%, and if any data is missing, the average value of the two days before and after should be used to fill it in. For outliers, traceability verification should be conducted, and they should be revisedby using the average value of the two days before and after unless there is a special anomaly. Overall, the quality of the meteorological data was good.

### 2.3 Statistical analysis

#### 2.3.1 Correlation analysis and SEM

According to the nonlinear effect of environmental factors on health, Spearman’s rank correlation coefficient was used to assess the association among air pollution and meteorological conditions. The collected meteorological data indicated that the warm season spans from May to October annually, while the cold season extends from November to April each year. Furthermore, structural equation modeling (SEM) was employed as an initial exploration tool for investigating both direct and indirect effects of environmental temperature and pollutants on mortality. SEM is a widely recognized statistical method capable of efficiently and promptly revealing relationships among latent variables or between latent variables and other variables. Although this analysis does not capture nonlinear effects, SEM can serve as a foundation for developing time series models [13]. In exploring the relationship between RD mortality toll and various latent environmental variables under complex environmental conditions, DMT was used as an intermediary. The basic structural equation model is presented below:

η=α+ΓX+δ

In the above equation, η represents the dependent variable; α and δ denote the intercept and residual, respectively; Γ signifies the coefficient for linear effects, and X denotes the latent variables (DMT, PM_2.5_, PM_10_, SO_2_, NO_2_, O_3_ and CO).

#### 2.3.2 Time series analysis

To mitigate the influence of confounding factors and to investigate both short-term effects and hysteresis effects, the distributed lag non-linear model (DLNM) was employed. This approach enables a more precise evaluation of the relationship between environmental exposure and health outcomes [14]. Our over-dispersion test confirmed that a poisson distribution was suitable for modeling mortality data for individuals aged less than 14 years old in our DLNM model, while for other subgroups, a binomial distribution was employed (S1 Table and S1–S6 Figs). The combined effects of DMT and air pollutants on RD mortality were assessed using a generalized additive model (GAM) in conjunction with DLNM. Confounding factors such as long-term trends (LTT), days of the week effect and holidays (DOW) were controlled for in the model. Model fitness and selection freedom were evaluated through the Akaike Information Criterion (AIC) and residual analysis. These methods allow for an assessment of nonlinear and lag effects resulting from combined environmental exposure on RD mortality. The procedure consisted of three steps: 1) establishing a basis cross matrix to identify the minimum mortality risk temperature (MMT), 2) constructing a model based on this basis cross matrix with MMT serving as the reference temperature, 3) adjusting dependent variables and degrees of freedom to make model predictions separately. The fundamental model can be expressed as follows:

$$G_{t}∼poisson/negative\;binomial(\delta_{t})$$

$$log(\delta_{t})=\alpha +\beta {DMT}_{t,l}+ns(RH_{t},df_{1})+ns(pollutants_{t},df_{2})+ns(Time_{t},7\times year)+DOW_{t}$$

In the above equation, *G*_*t*_ represents the actual number of mortalities on day t, while *δ*_*t*_ denotes the expected number of mortalities on day t. The intercept (*α*) and DMT matrix coefficients (*β*) represent the model parameters. The observation time is denoted by *t*, and *l* stands for lag days. The natural spline function is represented by ns(); *df*_1_ and *df*_2_ stand for the degrees of freedom (df) of RH and pollutants (PM_2.5_, PM_10_, SO_2_, NO_2_, O_3_ and CO), respectively. We calculated the relative risk (RR) and 95% confidence interval (CI), determined the reference temperature corresponding to the lowest RR value, and compared it with both minimum temperature (MinT) and maximum temperature (MaxT) values from DMT data. Additionally, we used excess risk (ER) along with its 95% CI to represent specific lag effects as well as cumulative risks associated with RD mortality per every 10 unit increase in ambient pollutant concentration [15]. To ensure reliability of our results in relation to air pollutants’ health effects, we gradually adjusted other pollutants within our models. As these effects may not be immediately observable but rather delayed or persistent over time periods, different lag structures were employed to estimate both lag-specific effects and cumulative lag effects using single-day lags (from lag0 to lag14) as well as several-day moving averages (from lag0-1 to lag0-14). By altering hysteresis modes such as single, distributed or limited distributed mode, we observed changes in shape of model’s hysteresis effect, which further enhanced the reliability of our estimation results.

#### 2.3.3 Stratification and sensitivity analysis

Stratification analysis was conducted to further investigate the expose-response-lag effect and examine the impact of gender, age and season. Sensitivity analysis was performed to evaluate the robustness of the model. The *dfs* in the temperature model were adjusted, including RH (*df*_1_ = 3 ~ 5) and pollutants (*df*_2_ = 3 ~ 5). Simultaneously, adjustments were made to df (*df*_1_~*df*_3_ = 3 ~ 5) in the pollutant model. For an additional sensitivity analysis, the study period was limited to 2014–2019. This exclusion of the potential impact of COVID-19 on air pollutant levels allowed us to assess the robustness of our findings and ensure the reliability of the results.

### 2.4 Software used

R software (version 4.2.2, www.r-project.org) was utilized for all statistical analyses. The “DLNM” and “spline” packages were used for fitting the temperature and pollutant models. The "lavaan" package was employed for SEM analysis of variables, and the "PerformanceAnalytics" package was used for correlation analysis. All statistical tests were two-tailed, with a significance level set at *p* <0.05.

## 3. Results

### 3.1 Descriptive statistics

Table 1 presents the distribution of RD mortalities, major ambient pollutants, and meteorological conditions in Qingdao from 2014 to 2020 (a total of 2557 days). A comprehensive collection of 19905 RD mortalities was obtained, with an average daily mortality rate of 7.78. Analysis revealed a higher incidence of daily mortalities among males (4.63) compared to females (3.15). Additionally, there was a higher incidence among the elderly(≥65 years old, 5.47) (5.47) compared to adults aged between 15 and 64 years old (2.25), and a very low incidence among children aged ≤14 years old (0.08).

**Table 1:** Summary of descriptive statistics for daily mortality rates, meteorological conditions, and daily air pollutants in Qingdao from 2014 to 2020 (2557 days).

Variables	N(%)	Mean±SD	Percentiles				
			Min	P25	P50	P75	Max
**RD**							
Total	19905(100)	7.78±4.35	0	5	7	10	45
Male	11840(59.5)	4.63±3.02	0	3	4	6	43
Female	8065(40.5)	3.15±2.24	0	2	3	4	15
≥65 years	13993(70.2)	5.47±3.50	0	3	5	7	25
15 ~ 64 years	5751(28.9)	2.25±2.51	0	2	2	3	40
≤14 years	161(0.9)	0.06±0.27	0	0	0	0	3
**Meteorological conditions**							
DMT(°C)	/	13.76±9.74	-12	5	15	23	31
DTR(°C)	/	8.04±2.81	1	6	8	10	19
RH(%)	/	69.35±15.22	28	58	70	81	100
P(mm)	/	1.74±7.07	0	0	0	0	151
WS(m/s)	/	2.73±1.00	1	2	3	3	7
AP(hpa)	/	1010.39±9.78	857	1002	1011	1018	1035
**Air pollutants**							
PM_2.5_(μg/m^3^)	/	43.98±30.59	5	24	35	54	258
PM_10_(μg/m^3^)	/	81.43±44.84	13	51	70	100	422
SO_2_(μg/m^3^)	/	18.40±16.33	4	8	13	18	196
NO_2_(μg/m^3^)	/	31.49±13.60	4	21	29	39	97
O_3_(μg/m^3^)	/	101.95±37.87	21	73	97	127	271
CO(mg/m^3^)	/	0.77±0.39	0.2	0.5	0.7	0.9	3.2

SD, standard deviation; Min, minimum; Max, maximum; RD, respiratory disease; DMT, daily mean temperature; DTR, diurnal temperature ranges; RH, relative humidity; P, precipitation; WS, wind speed; AP, air pressure; PM_2.5_ and PM_10,_ particulate matter less than 2.5 μm and less than 10 μm in aerodynamic diameter; SO_2_, sulfur dioxide; NO_2_, nitrogen dioxide; O_3_, ozone; CO, carbonic oxide.

PM_2.5_ and PM_10_ were found to be the predominant pollutants during the cold season, while O_3_ dominated in the warm season (S7 Fig). The annual average concentrations (standard deviation, SD) of PM_2.5_ in Qingdao from 2014 to 2020 was 43.98 (30.59) μg/m^3^ and the average concentration (SD) of PM_10_ was 81.43 (44.84) μg/m^3^. The average concentration (SD) of O_3_ was 101.95 (37.87) μg/m^3^.

### 3.2. Association between meteorological conditions, air pollutants, and RD mortality

**Fig 1:**
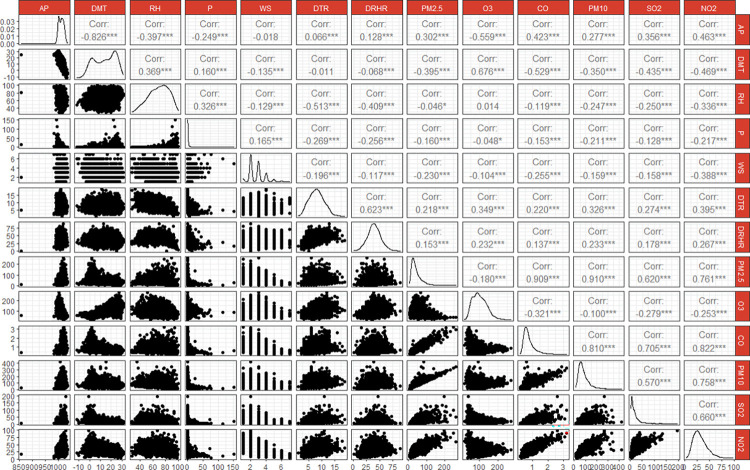
Spearman’s correlation coefficients between meteorological conditions and atmospheric pollutants. *P < 0.1; **P <0.05; ***P <0.01; Spearman’s correlation coefficients at the top, distribution plot at the middle and scatter plot at the bottom.

**Fig 2:**
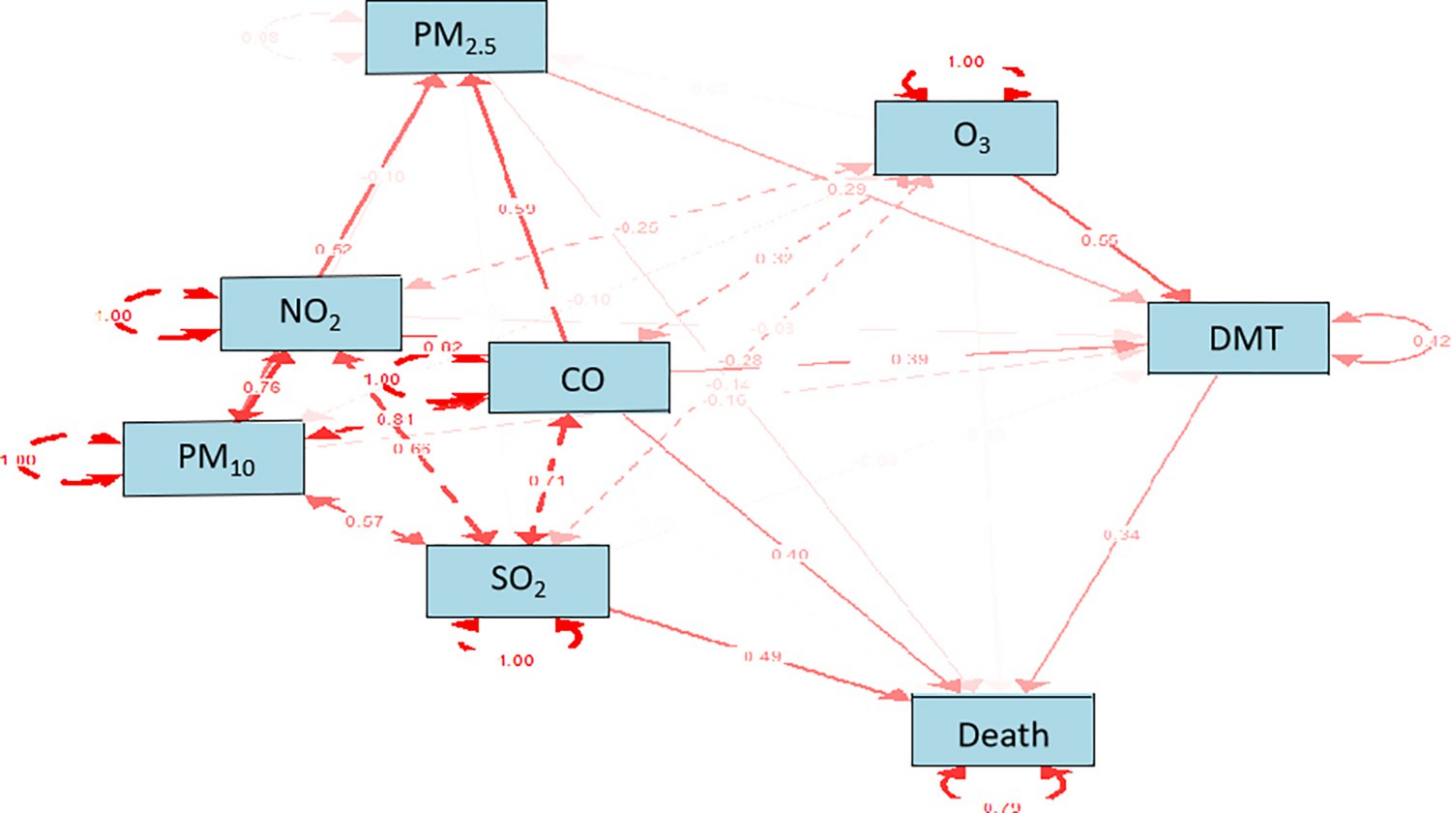
A structural equation modeling (SEM) analysis for direct and indirect effects mediated by DMT. A small effect was defined as less than 0.1, a moderate effect as approximately 0.3 and a significant effect as greater than 0.5.

The Spearman correlation between meteorological conditions and air pollutants is illustrated in Fig 1. DMT exhibits a positive correlation with O_3_ (r = 0.676, *p* <0.01), while displaying negative correlations with other pollutants, with r ranging from -0.350 to -0.529, *p* <0.01. Conversely, DTR shows positive correlations with all pollutants (*p* <0.01). AP demonstrates a negative correlation with DMT (r = - 0.826, *p* <0.01) and a positive correlation with DTR (r = 0.066, *p* <0.01). RH is positively correlated with DMT (r = 0.369, *p* <0.01) and negatively correlated with DTR (r = - 0.513, *p* <0.01). O_3_ exhibits negative correlations with all pollutants, whereas the remaining pollutants display significantly positive correlations among each other. The SEM model reveals that RD mortality is directly influenced by DMT, PM_2.5_, SO_2_ and CO; however, the other pollutants exert indirect effects on RD mortality as shown in Fig 2.

### 3.3. Cumulative risk of temperature on RD daily mortality

**Fig 3:**
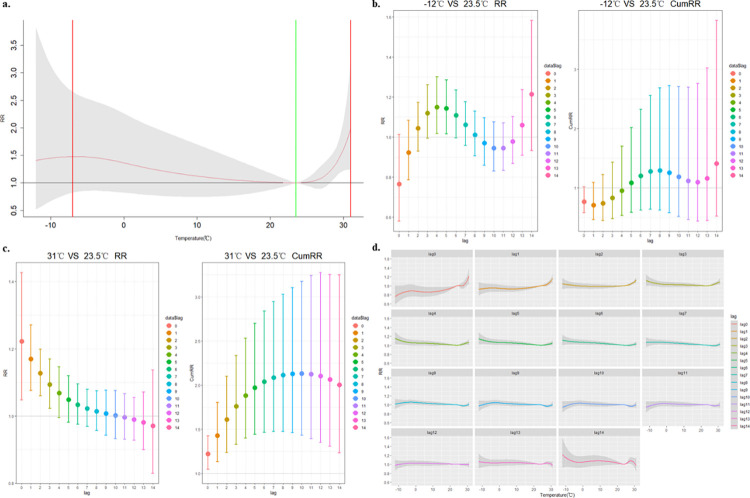
Minimum temperature, lag effects on RD mortality based on MMT. a, Total temperature effect; b, Minimum temperature(-12°C); c, Maximum temperature (31°C); d, daily effects from lag0 to lag14.

The model was developed with the MMT as a reference to enhance the comprehensibility and interpretability of the analysis results. DMT indicated both immediate short-term effects and lag effects on RD mortality (Fig 3). A strong correlation between daily RD mortality and DMT was observed, with the RR showing an increase when DMT decreased or increased relative to 23.5°C. Notably, we identified an N-shaped curve for the relationship between DMT and RD mortality, with 23.5°C representing the temperature associated with minimum mortality (Fig 3A).

The maximum RR at -12°C was observed on lag4 day (RR = 1.150, 95% CI: 1.017 ~ 1.301) and this single-day lag persisted for a duration of 2 days (Fig 3B). In the DMT range from -12°C to 23.5°C, a decrease in temperature was associated with an increase in RD daily mortality. The most significantly harmful effect was observed at 31°C on the lag0-8days (RR = 2.114, 95% CI: 1.475 ~ 3.028) (Fig 3C). The lagged analysis revealed that the maximum harmful effect occurred on lag0 day with an RR of 1.224 (95% CI: 1.047 ~ 1.427).

### 3.4 Excess risk of major pollutants on RD daily mortality

#### 3.4.1 Single-pollutant model

The GAM was used to investigate the effect of PM and O_3_ on RD daily mortality, revealing significant associations between pollutants and RD mortality. In the single-pollutant models, a 10 μg/m^3^ increase in PM_2.5_ exposure on lag1 day exhibited the highest risk of RD mortality, with an ER of 0.847% (95% CI: 0.335% ~ 1.362%). The largest cumulative effect was observed at a lag of 8 days, with an ER of 1.546% (95% CI: 0.483% ~ 2.621%). Similarly, for PM_10_ exposure, the highest risk occurred on lag0 day per 10 μg/m^3^ increase, yielding an ER of 0.531% (95% CI: 0.165% ~ 0.899%), while the largest cumulative effect was observed at a lag of 8 days with an ER of 1.110% (95% CI: 0.505% ~ 1.718%). Furthermore, for O_3_ exposure, the highest risk occurred on lag1 day per 10 μg/m^3^ increase, resulting in an ER (95% CI) of 1.073% (0.502% ~ 1.647%). The largest cumulative effect was observed at a lag of 2 days (ER = 1.113%, 95% CI: 0.386% ~ 1.844%) (Table 2).

**Table 2:** ER and 95%CI for the risk difference in RD daily mortality, using a single-pollutant model.

Lag days	PM_2.5_		PM_10_		O_3_	
	ER	95%CI	ER	95%CI	ER	95%CI
lag0	0.540	(-0.013, 1.095)	0.531	(0.165, 0.899)	0.340	(-0.276, 0.959)
lag1	0.847	(0.335, 1.362)	0.509	(0.160, 0.860)	1.073	(0.502, 1.647)
lag2	0.678	(0.169, 1.189)	0.389	(0.042, 0.736)	0.659	(0.117, 1.205)
lag3	0.499	(-0.013, 1.015)	0.400	(0.053, 0.748)	0.242	(-0.291, 0.779)
lag4	0.293	(-0.223, 0.812)	0.299	(-0.050, 0.649)	-0.093	(-0.625, 0.442)
lag5	0.529	(0.013, 1.048)	0.485	(0.137, 0.834)	-0.512	(-1.042, 0.021)
lag6	0.354	(-0.164, 0.875)	0.302	(-0.048, 0.653)	-0.618	(-1.149, -0.085)
lag7	0.328	(-0.192, 0.851)	0.135	(-0.217, 0.488)	-0.580	(-1.110, -0.046)
lag8	0.220	(-0.301, 0.744)	0.182	(-0.169, 0.535)	-0.771	(-1.301, -0.239)
lag9	-0.106	(-0.628, 0.420)	0.088	(-0.265, 0.443)	-0.523	(-1.055, 0.011)
lag10	-0.137	(-0.661, 0.389)	0.004	(-0.351, 0.361)	-0.640	(-1.173, -0.105)
lag11	0.019	(-0.503, 0.543)	-0.003	(-0.357, 0.352)	-0.763	(-1.298, -0.226)
lag12	0.159	(-0.362, 0.683)	0.012	(-0.342, 0.367)	-0.428	(-0.962, 0.108)
lag13	0.589	(0.070, 1.111)	0.213	(-0.140, 0.568)	-0.722	(-1.253, -0.187)
lag14	0.470	(-0.049, 0.992)	0.153	(-0.200, 0.506)	-0.925	(-1.453, -0.393)
lag0	0.540	(-0.013, 1.095)	0.531	(0.165, 0.899)	0.340	(-0.276, 0.959)
lag0-1	0.890	(0.293, 1.490)	0.655	(0.255, 1.057)	0.972	(0.288, 1.660)
lag0-2	1.071	(0.421, 1.724)	0.724	(0.289, 1.162)	1.113	(0.386, 1.844)
lag0-3	1.188	(0.482, 1.898)	0.818	(0.348, 1.291)	1.041	(0.276, 1.812)
lag0-4	1.254	(0.494, 2.020)	0.895	(0.391, 1.401)	0.864	(0.061, 1.673)
lag0-5	1.408	(0.598, 2.225)	1.031	(0.497, 1.568)	0.568	(-0.269, 1.412)
lag0-6	1.476	(0.631, 2.329)	1.089	(0.531, 1.651)	0.281	(-0.587, 1.157)
lag0-7	1.540	(0.656, 2.432)	1.090	(0.507, 1.676)	0.053	(-0.843, 0.957)
lag0-8	1.563	(0.644, 2.490)	1.110	(0.505, 1.718)	-0.215	(-1.134, 0.713)
lag0-9	1.459	(0.509, 2.417)	1.088	(0.462, 1.717)	-0.374	(-1.317, 0.578)
lag0-10	1.347	(0.369, 2.334)	1.047	(0.402, 1.696)	-0.548	(-1.513, 0.426)
lag0-11	1.342	(0.343, 2.351)	1.037	(0.377, 1.702)	-0.748	(-1.734, 0.248)
lag0-12	1.346	(0.324, 2.379)	1.010	(0.332, 1.692)	-0.828	(-1.834, 0.190)
lag0-13	1.453	(0.412, 2.506)	1.031	(0.338, 1.729)	-0.991	(-2.015, 0.043)
lag0-14	1.546	(0.483, 2.621)	1.048	(0.339, 1.762)	-1.201	(-2.24, -0.151)

#### 3.4.2 Dual-pollutant model

Based on the results of the single-pollutant model, we constructed two-pollutant models. Due to their high correlation, PM_2.5_ and PM_10_ were not simultaneously included in the model. The composite-pollutant model indicated that there still exited a slight increase in the effect of PM on the risk of RD mortality. In the two-pollutant models constructed with PM_2.5_ and CO, NO_2_, SO_2_ and O_3_, respectively, the maximum single-day lag ER occurred at lag8 (ER 1.641%, 1.271%, 1.201%, 1.103%, respectively). The maximum cumulative lag ER was observed in lag0-8 days (ER 1.059%, 1.641%, 1.271%, 1.562%, respectively). Similar trends were observed for models constructed with PM_10_ and CO, NO_2_, SO_2_ and O_3_ respectively (Table 3).

**Table 3:** ER and 95%CI for RD daily mortality, using composite-pollutant models based on PM_2.5_ and PM_10_.

Lag days	PM_2.5_+CO		PM_2.5_+NO_2_		PM_2.5_+SO_2_		PM_2.5_+O_3_	
	ER	95%CI	ER	95%CI	ER	95%CI	ER	95%CI
lag0	0.717	(-0.111, 1.552)	0.133	(-0.516, 0.788)	-1.223	(-2.564,0.136)	0.517	(0.149,0.886)
lag1	1.145	(0.374, 1.922)	0.619	(-0.036, 1.279)	0.551	(-0.502,1.615)	0.640	(0.239,1.044)
lag2	1.248	(0.475, 2.027)	0.834	(0.146, 1.526)	0.810	(-0.111,1.739)	0.711	(0.274,1.15)
lag3	1.319	(0.510, 2.135)	0.952	(0.214, 1.696)	0.900	(-0.003,1.811)	0.807	(0.335,1.28)
lag4	1.355	(0.498, 2.219)	1.003	(0.209, 1.802)	0.928	(0.007,1.856)	0.884	(0.380,1.391)
lag5	1.516	(0.611, 2.429)	1.148	(0.303, 2.000)	1.093	(0.139,2.055)	1.022	(0.488,1.559)
lag6	1.581	(0.641, 2.530)	1.205	(0.323, 2.095)	1.151	(0.170,2.141)	1.082	(0.524,1.644)
lag7	1.635	(0.658, 2.621)	1.260	(0.340, 2.188)	1.201	(0.188,2.223)	1.084	(0.501,1.67)
lag8	1.641	(0.632, 2.659)	1.271	(0.317, 2.235)	1.201	(0.158,2.256)	1.103	(0.498,1.712)
lag9	1.499	(0.460, 2.549)	1.149	(0.164, 2.143)	1.041	(-0.034,2.127)	1.081	(0.456,1.711)
lag10	1.357	(0.289, 2.437)	1.010	(-0.006, 2.035)	0.876	(-0.226,1.990)	1.042	(0.397,1.691)
lag11	1.347	(0.254, 2.451)	0.978	(-0.064, 2.031)	0.853	(-0.271,1.990)	1.033	(0.373,1.698)
lag12	1.344	(0.227, 2.474)	0.965	(-0.103, 2.045)	0.842	(-0.306,2.002)	1.006	(0.328,1.689)
lag13	1.463	(0.328, 2.611)	1.074	(-0.015, 2.174)	0.964	(-0.203,2.146)	1.028	(0.335,1.726)
lag14	1.561	(0.407, 2.727)	1.167	(0.057, 2.289)	1.069	(-0.118,2.270)	1.047	(0.338,1.761)
lag0	-1.338	(-2.488, -0.175)	0.717	(-0.111,1.552)	0.133	(-0.516,0.788)	0.511	(0.046,1.071)
lag0-1	0.237	(-0.696, 1.178)	1.145	(0.374,1.922)	0.619	(-0.036,1.279)	0.866	(0.266,1.469)
lag0-2	0.588	(-0.272, 1.454)	1.248	(0.475,2.027)	0.834	(0.146,1.526)	1.049	(0.398,1.704)
lag0-3	0.725	(-0.139, 1.597)	1.319	(0.510,2.135)	0.952	(0.214,1.696)	1.170	(0.464,1.881)
lag0-4	0.775	(-0.120, 1.679)	1.355	(0.498,2.219)	1.003	(0.209,1.802)	1.240	(0.479,2.006)
lag0-5	0.943	(0.007, 1.888)	1.516	(0.611,2.429)	1.148	(0.303,2.000)	1.399	(0.589,2.216)
lag0-6	1.001	(0.032, 1.979)	1.581	(0.641,2.530)	1.205	(0.323,2.095)	1.473	(0.627,2.326)
lag0-7	1.053	(0.051, 2.065)	1.635	(0.658,2.621)	1.260	(0.340,2.188)	1.539	(0.654,2.431)
lag0-8	1.059	(0.029, 2.100)	1.641	(0.632,2.659)	1.271	(0.317,2.235)	1.562	(0.644,2.489)
lag0-9	0.907	(-0.152, 1.976)	1.499	(0.460,2.549)	1.149	(0.164,2.143)	1.458	(0.509,2.417)
lag0-10	0.743	(-0.344, 1.842)	1.357	(0.289,2.437)	1.010	(-0.006,2.035)	1.349	(0.371,2.336)
lag0-11	0.715	(-0.396, 1.838)	1.347	(0.254,2.451)	0.978	(-0.064,2.031)	1.347	(0.347,2.356)
lag0-12	0.699	(-0.437, 1.848)	1.344	(0.227,2.474)	0.965	(-0.103,2.045)	1.351	(0.329,2.384)
lag0-13	0.818	(-0.339, 1.987)	1.463	(0.328,2.611)	1.074	(-0.015,2.174)	1.459	(0.417,2.512)
lag0-14	0.921	(-0.256, 2.111)	1.561	(0.407,2.727)	1.167	(0.057,2.289)	1.553	(0.490,2.628)
Lag days	PM_10_+CO		PM_10_+NO_2_		PM_10_+SO_2_		PM_10_+O_3_	
	ER	95%CI	ER	95%CI	ER	95%CI	ER	95%CI
lag0	-1.338	(-2.488, -0.175)	0.349	(-0.063,0.764)	0.061	(-0.561,0.687)	0.803	(0.281,1.329)
lag1	0.237	(-0.696, 1.178)	0.501	(0.071,0.932)	0.336	(-0.225,0.900)	0.839	(0.333,1.348)
lag2	0.588	(-0.272, 1.454)	0.584	(0.129,1.040)	0.431	(-0.109,0.974)	0.834	(0.321,1.349)
lag3	0.725	(-0.139, 1.597)	0.683	(0.197,1.171)	0.544	(-0.005,1.097)	0.904	(0.369,1.443)
lag4	0.775	(-0.120, 1.679)	0.756	(0.237,1.277)	0.624	(0.053,1.198)	0.970	(0.406,1.537)
lag5	0.943	(0.007, 1.888)	0.891	(0.342,1.443)	0.775	(0.179,1.374)	1.117	(0.525,1.713)
lag6	1.001	(0.032, 1.979)	0.945	(0.372,1.522)	0.829	(0.210,1.450)	1.172	(0.556,1.791)
lag7	1.053	(0.051, 2.065)	0.941	(0.344,1.542)	0.815	(0.176,1.458)	1.156	(0.517,1.798)
lag8	1.059	(0.029, 2.100)	0.957	(0.338,1.579)	0.827	(0.170,1.489)	1.165	(0.506,1.828)
lag9	0.907	(-0.152, 1.976)	0.931	(0.292,1.573)	0.791	(0.115,1.472)	1.128	(0.450,1.812)
lag10	0.743	(-0.344, 1.842)	0.880	(0.221,1.543)	0.731	(0.036,1.432)	1.075	(0.376,1.779)
lag11	0.715	(-0.396, 1.838)	0.855	(0.178,1.536)	0.707	(-0.004,1.424)	1.060	(0.343,1.781)
lag12	0.699	(-0.437, 1.848)	0.817	(0.122,1.517)	0.663	(-0.067,1.399)	1.023	(0.287,1.763)
lag13	0.818	(-0.339, 1.987)	0.833	(0.123,1.549)	0.678	(-0.069,1.429)	1.042	(0.292,1.798)
lag14	0.921	(-0.256, 2.111)	0.846	(0.119,1.578)	0.690	(-0.071,1.457)	1.056	(0.29,1.828)
lag0	0.061	(-0.561,0.687)	0.803	(0.281,1.329)	0.349	(-0.063,0.764)	0.517	(0.149,0.886)
lag0-1	0.336	(-0.225,0.900)	0.839	(0.333,1.348)	0.501	(0.071,0.932)	0.640	(0.239,1.044)
lag0-2	0.431	(-0.109,0.974)	0.834	(0.321,1.349)	0.584	(0.129,1.04)	0.711	(0.274,1.150)
lag0-3	0.544	(-0.005,1.097)	0.904	(0.369,1.443)	0.683	(0.197,1.171)	0.807	(0.335,1.280)
lag0-4	0.624	(0.053,1.198)	0.970	(0.406,1.537)	0.756	(0.237,1.277)	0.884	(0.380,1.391)
lag0-5	0.775	(0.179,1.374)	1.117	(0.525,1.713)	0.891	(0.342,1.443)	1.022	(0.488,1.559)
lag0-6	0.829	(0.210,1.450)	1.172	(0.556,1.791)	0.945	(0.372,1.522)	1.082	(0.524,1.644)
lag0-7	0.815	(0.176,1.458)	1.156	(0.517,1.798)	0.941	(0.344,1.542)	1.084	(0.501,1.670)
lag0-8	0.827	(0.170,1.489)	1.165	(0.506,1.828)	0.957	(0.338,1.579)	1.103	(0.498,1.712)
lag0-9	0.791	(0.115,1.472)	1.128	(0.450,1.812)	0.931	(0.292,1.573)	1.081	(0.456,1.711)
lag0-10	0.731	(0.036,1.432)	1.075	(0.376,1.779)	0.880	(0.221,1.543)	1.042	(0.397,1.691)
lag0-11	0.707	(-0.004,1.424)	1.060	(0.343,1.781)	0.855	(0.178,1.536)	1.033	(0.373,1.698)
lag0-12	0.663	(-0.067,1.399)	1.023	(0.287,1.763)	0.817	(0.122,1.517)	1.006	(0.328,1.689)
lag0-13	0.678	(-0.069,1.429)	1.042	(0.292,1.798)	0.833	(0.123,1.549)	1.028	(0.335,1.726)
lag0-14	0.690	(-0.071,1.457)	1.056	(0.290,1.828)	0.846	(0.119,1.578)	1.047	(0.338,1.761)

#### 3.4.3 Full-pollutant model

The results of the full-pollutant model (Table 4), similar to those obtained from the single-pollutant and dual-pollutant models, demonstrated that PM still exerted an impact on the risk of RD mortality. Notably, for PM_2.5_ and PM_10_, the highest ER values were observed at lag1 day and lag5 day respectively, with corresponding estimates of 0.558% (0.033% ~ 1.087%) and 0.471% (0.120% ~ 0.823%), respectively. Furthermore, both pollutants exhibited their maximum cumulative effects in lag0-8 days, yielding ER estimates (95% CI) of 1.216% (0.182% ~ 2.260%) and 1.021% (0.354% ~ 1.691%), respectively.

**Table 4:** ER and 95%CI for RD daily mortality, using full-pollutant models based on PM_2.5_ and PM_10_.

Lag days	PM_2.5_+SO_2_+NO_2_+O_3_+CO		PM_10_+SO_2_+NO_2_+O_3_+CO	
	ER	95%CI	ER	95%CI
lag0	-1.212	(-2.400, -0.010)	0.294	(-0.350, 0.942)
lag1	0.576	(-0.025, 1.182)	0.366	(-0.031, 0.765)
lag2	0.558	(0.033, 1.087)	0.349	(-0.008, 0.707)
lag3	0.452	(-0.066, 0.973)	0.401	(0.050, 0.754)
lag4	0.249	(-0.271, 0.772)	0.295	(-0.057, 0.648)
lag5	0.477	(-0.044, 1.001)	0.471	(0.120, 0.823)
lag6	0.285	(-0.238, 0.810)	0.282	(-0.071, 0.635)
lag7	0.267	(-0.256, 0.792)	0.122	(-0.231, 0.477)
lag8	0.169	(-0.354, 0.694)	0.181	(-0.172, 0.535)
lag9	-0.176	(-0.701, 0.351)	0.080	(-0.275, 0.436)
lag10	-0.236	(-0.763, 0.294)	-0.020	(-0.378, 0.338)
lag11	-0.093	(-0.620, 0.437)	-0.037	(-0.393, 0.322)
lag12	0.041	(-0.485, 0.570)	-0.030	(-0.387, 0.328)
lag13	0.464	(-0.059, 0.989)	0.166	(-0.189, 0.522)
lag14	0.380	(-0.141, 0.903)	0.133	(-0.221, 0.488)
lag0	-1.212	(-2.400, -0.010)	0.294	(-0.350, 0.942)
lag0-1	0.345	(-0.608, 1.307)	0.507	(-0.069, 1.086)
lag0-2	0.708	(-0.166, 1.589)	0.592	(0.040, 1.147)
lag0-3	0.857	(-0.016, 1.738)	0.713	(0.153, 1.276)
lag0-4	0.914	(0.013, 1.823)	0.798	(0.217, 1.382)
lag0-5	1.092	(0.152, 2.042)	0.959	(0.354, 1.567)
lag0-6	1.153	(0.180, 2.136)	1.016	(0.389, 1.647)
lag0-7	1.210	(0.204, 2.226)	1.004	(0.357, 1.657)
lag0-8	1.216	(0.182, 2.260)	1.021	(0.354, 1.691)
lag0-9	1.065	(0.003, 2.139)	0.990	(0.305, 1.680)
lag0-10	0.899	(-0.192, 2.002)	0.932	(0.228, 1.642)
lag0-11	0.860	(-0.254, 1.987)	0.904	(0.183, 1.631)
lag0-12	0.839	(-0.300, 1.992)	0.861	(0.121, 1.606)
lag0-13	0.943	(-0.216, 2.115)	0.871	(0.116, 1.632)
lag0-14	1.034	(-0.145, 2.226)	0.883	(0.114, 1.659)

### 3.5 Stratified analysis

**Fig 4:**
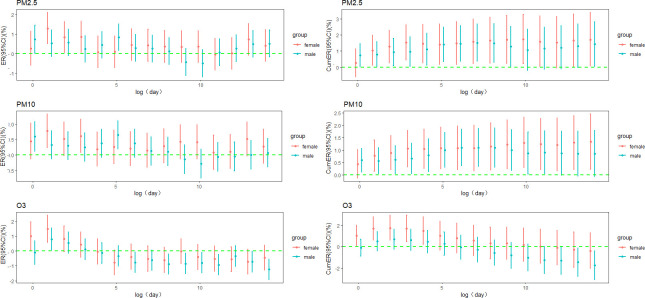
Sex-stratified lag and cumulative ER in RD mortality per 10 μg/m^3^ PM_2.5_, PM_10_, O_3_.

The females show a greater sensitivity to the risk of RD mortality compared to males. The highest ER per 10 μg/m^3^ increase in PM_2.5_ for females was observed at lag1 day (ER = 1.294%, 95%CI: 0.501% ~ 2.093%), while for males, it occurred at lag5 day (ER = 0.834%, 95%CI: 0.162% ~ 1.510%). Females also had the highest cumulative ER at lag0-10 days (ER = 1.742%, 95%CI: 0.224% ~ 3.282%), whereas males exhibited the highest cumulative ER at lag0-6 days (ER = 1.515%, 95%CI: 0.362% ~ 2.681%). Similar patterns were observed for PM_10_ and O_3_ (Fig 4).

**Fig 5:**
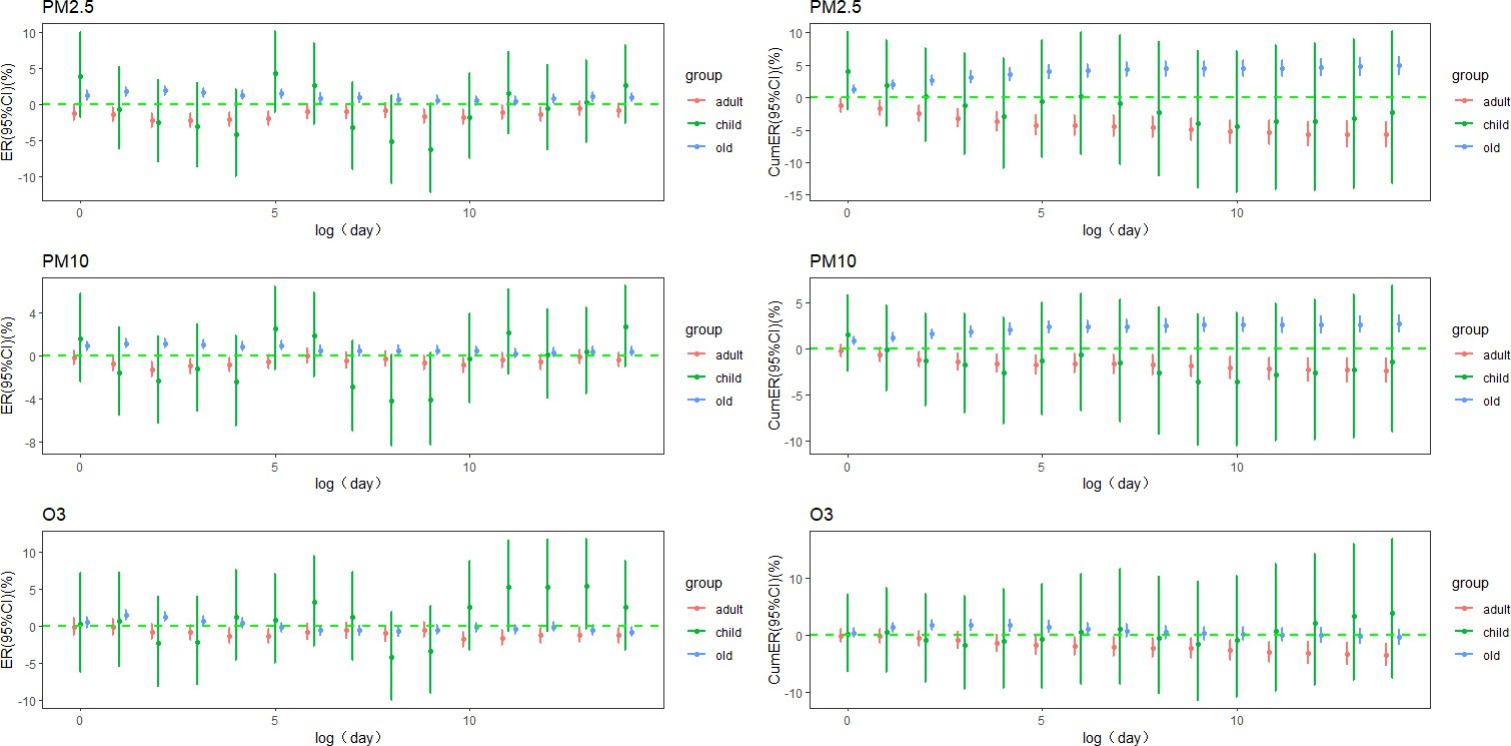
Age-stratified lag and cumulative ER in RD mortality per 10 μg/m^3^ PM_2.5_, PM_10_, O_3_.

In the elderly population, the RD mortality ER per 10 μg/m^3^ increase in PM_2.5_ reached its maximum at lag2 day (ER = 1.291%, 95%CI: 1.891% ~ 2.495%) and the cumulative ER reached its maximum at lag0-14 days (ER = 4.896%, 95%CI: 3.598% ~ 6.210%) No significant associations between ER or cumulative ER were found among children and adults. These trends were consistent with those observed for PM_10_ and O_3_ exposure (Fig 5).

**Fig 6:**
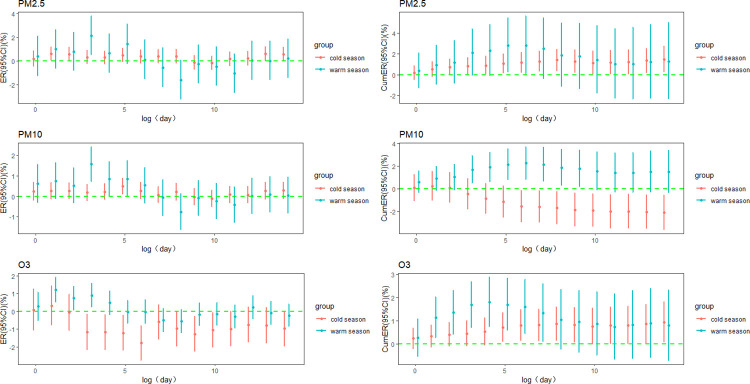
Season-stratified lag and cumulative ER in RD mortality per 10 μg/m^3^ PM_2.5_, PM_10_, O_3_.

In the warm season, the lag3 day ER per 10 μg/m^3^ increase in PM_2.5_ reached its maximum (ER = 2.132%, 95%CI: 0.530% ~ 3.761%), and the cumulative ER peaked at lag0-5 days (ER = 2.814%, 95%CI: 0.264% ~ 5.429%). Similar trends were observed for PM_10_ and O_3_ concentrations as well. However, in the cold season, both single-day ER and cumulative ER of O_3_ were higher compared to those in the warm season, with the highest ER per 10 μg/m^3^ increase in O_3_ occurring at lag0-14 days (ER = 0.940%, 95%CI: 0.094% ~ 1.793%) (Fig 6).

### 3.6 Sensitive analysis

As shown in S8 Fig, the construction of the DLNM involves selecting different parameters to investigate the impact of short-term air pollutants on health outcomes, leading to varying results. To assess model robustness, adjustments were made to the time degree of freedom controlling long-term trends within the natural cubic spline function. The range of yearly freedom variations ranged from 6 to 10, and each air pollutant’s health effects were estimated through fitting procedures. In the optimal lag period, while there was a slight change in the RR for each air pollutant’s association with RD mortality as time freedom increased, overall risk remained stable. This suggests that changes in time freedom had minimal effect on the air pollutant-related RD mortality. The estimates remained unchanged even from 2014–2019 (S8–S12 Figs), indicating the robustness of our results despite any potential COVID-19 impact.

## 4. Discussion

In this study, meteorological conditions (DMT) and air pollutants (PM_2.5_, PM_10_ and O_3_) were found to significantly impact the risk of RD mortality in the Qingdao population, particularly in females, the elderly and during warm seasons.

Firstly, we observed that temperature plays a crucial role in RD mortality in Qingdao. The cumulative lag effect of low temperature on RD persists longer than that of high temperature; however, the cumulative lag effect of high temperature is stronger than that of low temperature. Both lower DMT and greater DTR were observed to exacerbate the impact on RD mortality. The temperate maritime climate in Qingdao, with prolonged spring and autumn seasons and significant temperature differences between day and night, can cause increased respiratory irritation among individuals with underlying diseases when exposed to extreme hot or cold air combined with pollutants. Additionally, our findings indicate that RD mortality exhibits more statistical significance during warm seasons due to heating practices implemented during cold seasons in Qingdao. Cold prevention measures demonstrate certain protective effects on the respiratory tract [16–18].

Secondly, our study showed that O_3_ was the predominant pollutant during the warm season, while PM emerged as the primary pollutant during the cold season, with its concentration exerting a certain impact on the ER of RD in Qingdao. From 2014 to 2020, both PM_2.5_ and PM_10_ concentrations in Qingdao exceeded the standard for an alarming number of days (2394 and 2119 days, respectively), surpassing WHO’s recommended average daily levels of 15 μg/m^3^ for PM_2.5_ and 45 μg/m^3^ for PM_10_. Furthermore, annual average concentrations of both pollutants surpassed WHO’s first-level annual average concentration standards. The average annual concentration of O_3_ in Qingdao was found to be higher than the WHO peak-season limit of 60 μg/m^3^, and the daily moving average concentration exceeded the limit for 1221 days (WHO daily AQG-level O_3_-8h was 100 μg/m^3^). China exhibits a high level of particulate matter exposure; however, studies conducted in China regarding PM_2.5_ and population mortality risk have reported lower correlation effect values compared to those observed in Europe and the US [19, 20]. Chen et.al demonstrated that across 272 Chinese cities, the mean annual concentration of PM_2.5_ was recorded at 56 μg/m^3^, far exceeding WHO’s air quality guidelines (annual mean: 10 μg/m^3^). Additionally, they documented a daily average death toll from RD at approximately two cases [21]. Notably, there were significant variations in climatic conditions among these cities. In our study specifically focused on Qingdao city, we found that the annual mean concentration of PM_2.5_ averaged at 44 μg/m^3^ alongside an average daily death toll from RD reaching approximately 7.8 cases per day——a figure significantly surpassing national averages warranting attention.

Although there is a wealth of evidence linking PM_2.5_ and O_3_ to human health outcomes [22, 23], previous studies have predominantly examined the health impacts of these pollutants in isolation, often overlooking potential interactive effects and treating the other pollutant merely as a confounding factor. Hence, we have developed a variety of pollutant models to assess the effects of both pollutants. Recent epidemiological evidence has suggested that PM and O_3_ may act as confounding factors in studies investigating the relationship between ambient temperature and mortality [6, 24]. Furthermore, some studies have also identified O_3_ as an effect modifier in warm seasons [16, 25]. In other words, the association between temperature and RD mortality is partially influenced by the effects of PM and O_3_. However, it should be noted that this confounding effect is relatively small, indicating that both temperature and air pollution independently contribute to mortality. This finding aligns with our own research results, which demonstrate that stratified analyses reveal distinct health effects of PM and O_3_ across different seasons. Additionally, when conducting a stratified analysis of pollutants under controlled meteorological conditions, we observed a significantly narrower confidence interval for O_3_ compared to PM. This stronger confidence level supports previous study findings. Previous studies have indicated that higher levels of O_3_ may lead to a greater impact on mortality when combined with temperature [16, 25]. The presence of strong correlations between pollutants and temperature poses challenges to distinguish the independent effects of each exposure, which could explain the conflicting evidence regarding confounding and effect modification by air pollutants. Further investigation with larger sample sizes is warranted to comprehensively understand the health effects of O_3_ on the population. Sara D et al. have reported suggestive evidence of increased morbidity and mortality associated with higher short-term PM_10-2.5_ concentrations, particularly for respiratory diseases compared to cardiovascular endpoints [26]. Correlation analysis shows a nearly linear relationship between PM_2.5_ and PM_10_, with similar magnitudes observed in single-day lag and cumulative lag based on the pollutant-model analysis. Liu et al. demonstrated that RD mortality rate was 0.41% (95%CI, 0.06% ~ 0.77%) per every 10 μg/m^3^ increase in PM_2.5_ concentrations on lag01 day; these associations remained robust after adjusting for all co-pollutants, especially PM_2.5_, in the two-pollutant model. The exposure-response curves for total and RD mortality were positive without discernible thresholds but steeper slopes at lower exposure ranges [27]. Our findings indicate that RD mortality rates were 0.847% (95%CI, 0.335% ~ 1.362%) and 0.531% (95%CI, 0.165% ~ 0.899%) per every 10 μg/m^3^ increase in PM_2.5_ and PM_10_ concentrations on lag01 day, respectively. Composite-pollutants-model results showed that the effect of PM on the risk of RD mortality still existed with slightly increased effect sizes; furthermore, composite pollutants had a higher risk effect than single pollutants did while reaching peak cumulative effects after one week of exposure duration. The full-pollutants-model results indicated that PM still exerted an effect on the risk of RD mortality. We hypothesize that the disparity in RD mortalities between Qingdao and the entire country may be attributed to variations in study areas and differential levels of exposure to air pollutants, resulting in heterogeneous health effects on humans. Consequently, it is imperative to conduct risk assessments of pollutants on RD mortality across multiple regions and perform model comparisons to ascertain whether PM_2.5_ and PM_10_ exert independent effects, thereby determining the necessity for distinct regulatory measures for each pollutant.

In addition, it has been observed that age modifies the association between temperature and mortality [16, 28–30]. Vulnerable groups, particularly individuals aged 65 years or older, infants and those with co-morbid cardiovascular and/or respiratory conditions, exhibit impaired thermoregulation. Consequently, when temperatures exceed a certain threshold during cold winter spells or heat waves, there is an elevated risk of mortality [31]. Our study reveals that females and the elderly in Qingdao display heightened sensitivity to climate change and air pollution due to multiple environmental factors. While statistical significance was found for RD mortality among the elderly population, no such significance was observed in children and adults. The results of stratified analysis showed a positive correlation between the width of the confidence interval and the level of confidence. This association can be attributed to our study’s focus on mortality data, with the elderly population accounting for 70.2% of the dataset. Our findings align with those reported by Gouveia et. al [32]. Conversely, Feng et al. [33] indicated that younger individuals (age < 65 years) and males may exhibit increased vulnerability to PM_2.5_ exposure, while the elderly face a higher cumulative risk of mortality. Yin et al. [23] have also confirmed that elevated PM_2.5_ concentrations are associated with higher rates of RD-related mortality in males. Despite females comprising a lower proportion (40.5%) of mortalities in our dataset compared to males, our study reveals their heightened sensitivity and vulnerability to changes in complex environmental factors. Further investigations are warranted to elucidate the precise reasons underlying these gender differences. Yu et al. [34] discovered that it is crucial for young individuals to acknowledge the heightened risk associated with exposure to exceedingly high RH and DTR. Qingdao, characterized by unique humidity conditions and significant day-night temperature fluctuations throughout the year, represents a focal point of our forthcoming investigation.

Our study possesses several strengths. Firstly, it is widely acknowledged that a longer study period is advantageous due to the relationship between sample content in time series analysis and two factors: time scale and study period. In the case of a fixed time scale, an extended study period results in larger sample size and more stable estimates of model parameters. To ensure quality control during data collection, we carefully selected relevant data from seven national meteorological observation stations and 25 air quality monitoring stations in Qingdao spanning from 2014 to 2020. We conducted necessary data processing such as replacing missing values and dimension reduction, which effectively represents exposure factors of Qingdao city while avoiding information bias and selection bias. Secondly, we considered six common air pollutants comprehensively by employing multi-pollutant models to assess the robustness of estimated associations between each pollutant and RD mortality. Thirdly, our study employed stratified analysis accounting for long-term trends as well as seasonal variations while controlling for individual-level confounding factors including sex and age. Notably, Qingdao’s unique geographical position within China with distinctive terrestrial and Marine climate conditions holds significant implications for investigating the impact of climate change along with complex environmental factors.

This study also has several limitations. Primarily, the application of DLNM combined with time series analysis falls within the realm of comparative ecology research in ecological studies. Its notable characteristic lies in its population-level basis, and it would be inappropriate to generalize it to an individual level without biological evidence substantiating such claims. This study sole focuses on the population level data from Qingdao City, thus precluding any inference regarding the impact of individual exposure levels arising from meteorological conditions and air pollution. Due to our study design, we cannot consider the biological component of ambient air and the geospatial distribution of PM. Similarly, we cannot account for factors such as access to medical resources, disadvantaged groups, and migrant population which may have influenced our findings. Furthermore, the daily mortalities of respiratory diseases were obtained from the Chronic Disease Surveillance Monitoring System in Qingdao, which does not guarantee complete information on all deaths. The lack of complete case data may lead to reduced accuracy in our results. Lastly, due to heterogeneity among various cities, it is difficult to generalize the results from a single city to other areas.

## 5. Conclusions

This study is the first investigation into the association between meteorological conditions, atmospheric pollutants and RD mortality risk in Qingdao, China. Our findings reveal that meteorological conditions (specifically DMT) and air pollutants (including PM_2.5_, PM_10_ and O_3_) significantly influence the risk of RD mortality in Qingdao’s population, especially among females, elderly individuals, and during warmer seasons. Notably, we observed an extreme cumulative risk of RD mortality during both high-temperature and low-temperature periods. As high temperatures gradually increased, so did the risk of RD mortality; moreover, this effect was more pronounced than that associated with low temperatures. Additionally, higher pollution levels coupled with longer lag time were found to elevate the risk of RD mortality. Specifically regarding particulate matter, we noted a larger effect size with increasing cumulative lag time. Throughout our monitoring period in Qingdao’s climate context, PM_2.5_ and PM_10_ emerged as primary pollutants during colder seasons while O_3_ dominated during warmer seasons. The impact of a single pollutant on RD mortality generally peaks on the day of exposure or one day thereafter; whereas the cumulative effect of multiple pollutants generally reaches its peak one week later. Furthermore, our results indicate that females and elderly individuals in Qingdao are more sensitive to climate change and air pollution under various environmental influences. Therefore, it is crucial to continue implementing sustainable development policies in Qingdao, which can serve as a foundation for assessing health risks associated with climate change and adapting population health strategies. Efforts should be intensified to enhance monitoring systems, early warning systems, prevention measures, and control strategies targeting climate-sensitive diseases such as respiratory ailments. Effective approaches should also be explored to safeguard public health amidst the dual challenges posed by carbon emissions.

## Supporting information

S1 Table Mortality distribution tests. (DOCX)

S1 Fig Model diagnostics for all death events. (TIF)

S2 Fig Model diagnostics for male death events. (TIF)

S3 Fig Model diagnostics for female death events. (TIF)

S4 Fig Model diagnostics for adult death events. (TIF)

S5 Fig Model diagnostics for old death events. (TIF)

S6 Fig Model diagnostics for child death events. (TIF)

S7 Fig Temporal distribution of primary pollutants in Qingdao from 2014 to 2020 based on a calendar map. a, PM_2.5_; b, PM_10_; c, O_3_.
(TIF)

S8 Fig Effect of atmospheric pollutants with varying degrees of freedom on the RD mortality rate (df = 7). (TIF)

S9 Fig Lagged effects of DMT on RD mortality in Qingdao from2014 to 2019. (TIF)

S10 Fig Determine the minimum temperature associated with mortality in Qingdao from2014 to 2019. (TIF)

S11 Fig The single lag effect and cumulative lag effect of extreme temperature on RD mortality based on MMT in Qingdao from2014 to 2019. a, Minimum temperature(-12°C); b, Maximum temperature (31°C).
(TIF)

S12 Fig Daily effects of DMT on RD mortality from lag0 to lag14. (TIF)
