# Supplementary material for: Short-term effects of combined environmental factors on respiratory disease mortality in Qingdao city: A time-series investigation
Source: PLoS One. 2025 Jan 28;20(1):e0318250. doi: 10.1371/journal.pone.0318250 (PMC11774373; doi:10.1371/journal.pone.0318250)
Supplement: S1 Table — (DOCX) [file pone.0318250.s001.docx]

S1_Table. Mortality distribution tests.

|  | Mean | Variance | Variance/Mean ratio | AIC (Poisson) | AIC (Negative Binomial) | Quasi-Poisson dispersion | Dispersion test p-value | Negative Binomial theta |
| --- | --- | --- | --- | --- | --- | --- | --- | --- |
| All | 7.78 | 18.92 | 2.4 | 15496.84 | 14267.72 | 2.43 | 1.15e-39 | 5.96 |
| Mall | 4.63 | 9.09 | 1.96 | 12877.07 | 12275.67 | 1.96 | 3.77e-11 | 5.57 |
| Female | 3.15 | 5.01 | 1.59 | 11126.46 | 10833.88 | 1.59 | 2.77e-30 | 5.60 |
| Old | 5.47 | 12.26 | 2.24 | 14134.36 | 13116.83 | 2.24 | 6.80e-62 | 4.59 |
| Adult | 2.25 | 6.32 | 2.81 | 11514.31 | 10178.41 | 2.81 | 2.08e-09 | 1.57 |
| Child | 0.06 | 0.07 | 1.19 | 1240.965 | 1219.48 | 1.19 | 0.002 | 0.33 |
