# Supplementary figures and images for: Short-term effects of combined environmental factors on respiratory disease mortality in Qingdao city: A time-series investigation
Source: PLoS One. 2025 Jan 28;20(1):e0318250. doi: 10.1371/journal.pone.0318250 (PMC11774373; doi:10.1371/journal.pone.0318250)

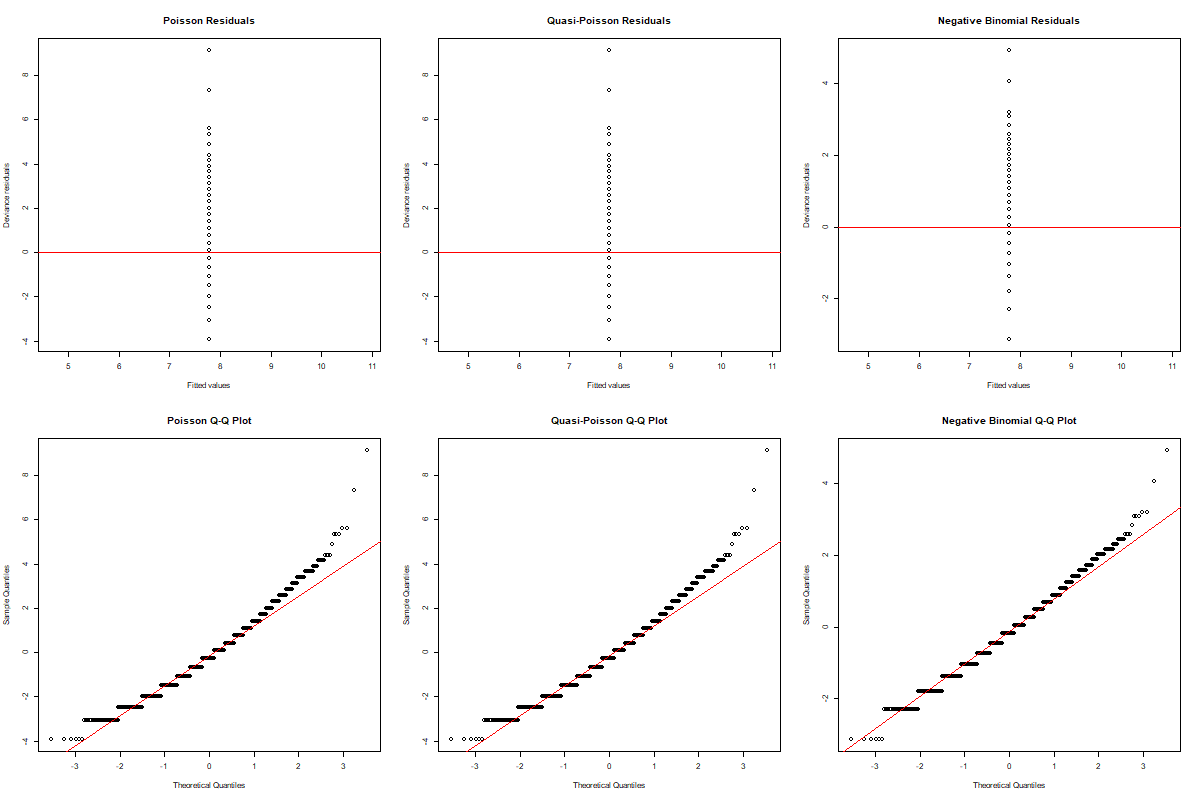

Supplement: S1 Fig — (TIF) [file pone.0318250.s002.tif]

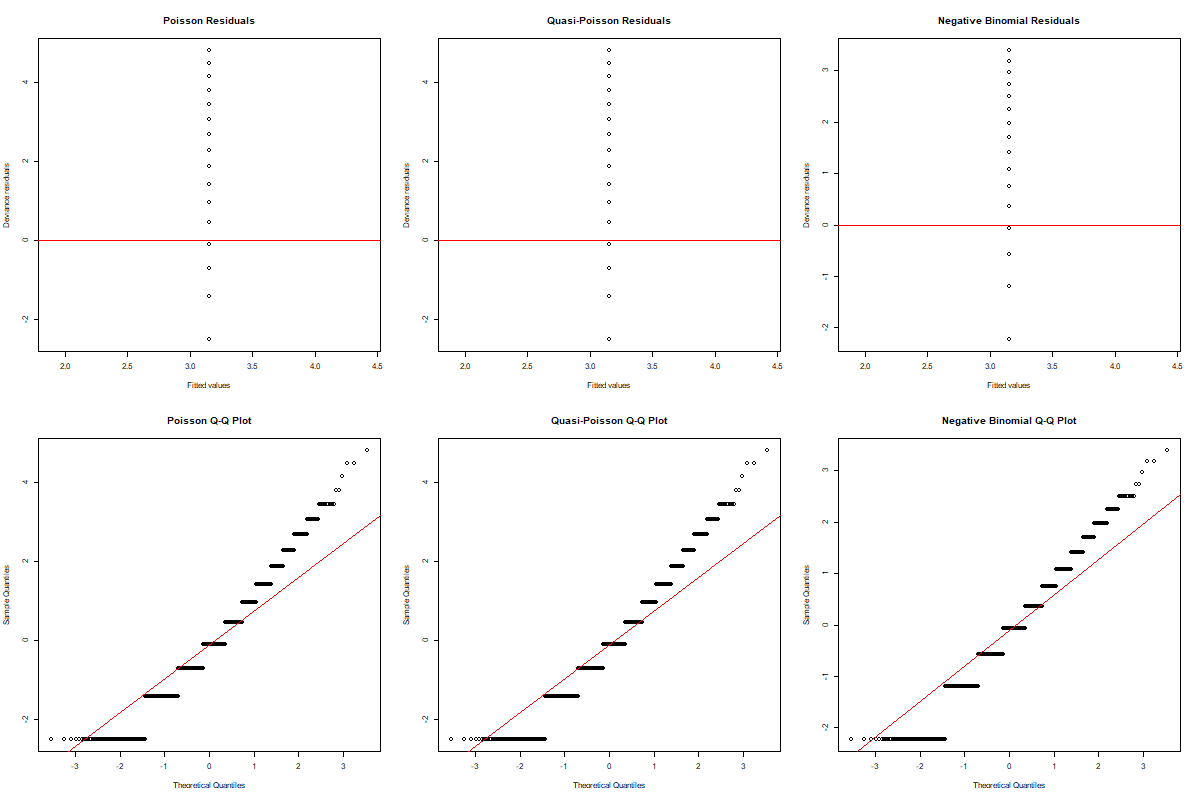

Supplement: S2 Fig — (TIF) [file pone.0318250.s003.tif]

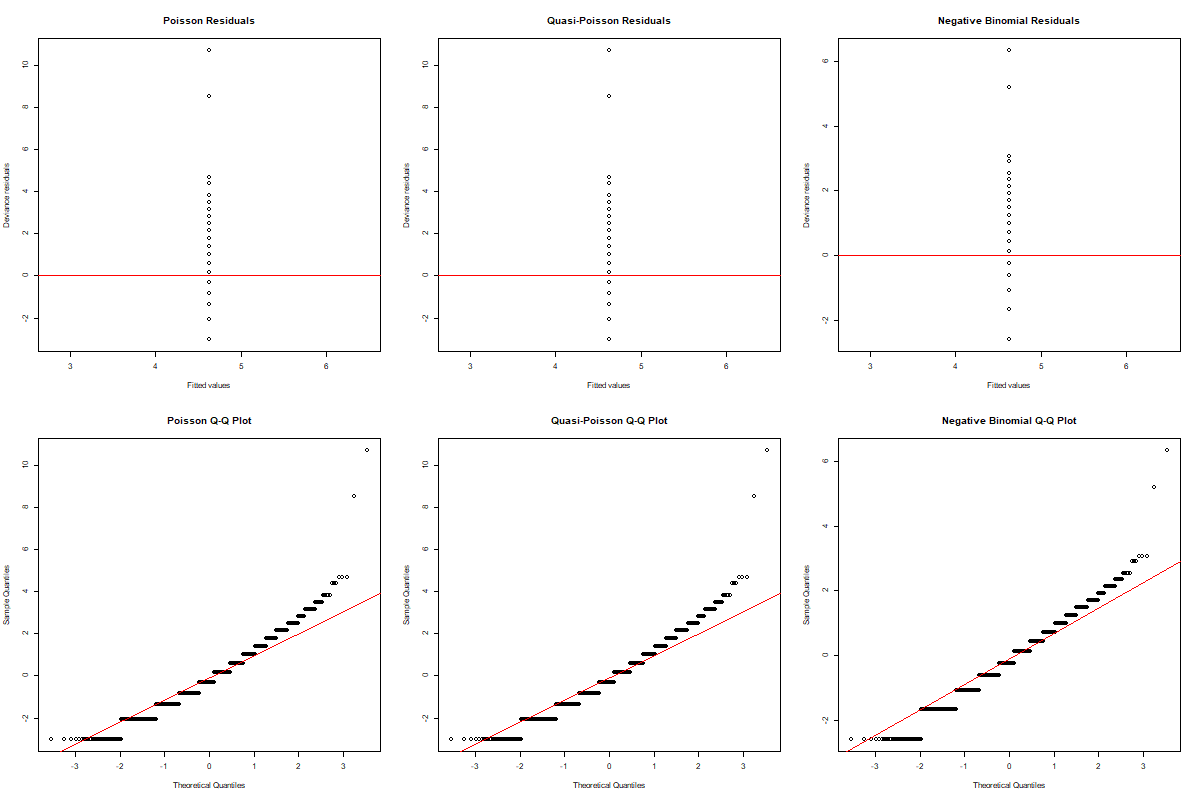

Supplement: S3 Fig — (TIF) [file pone.0318250.s004.tif]

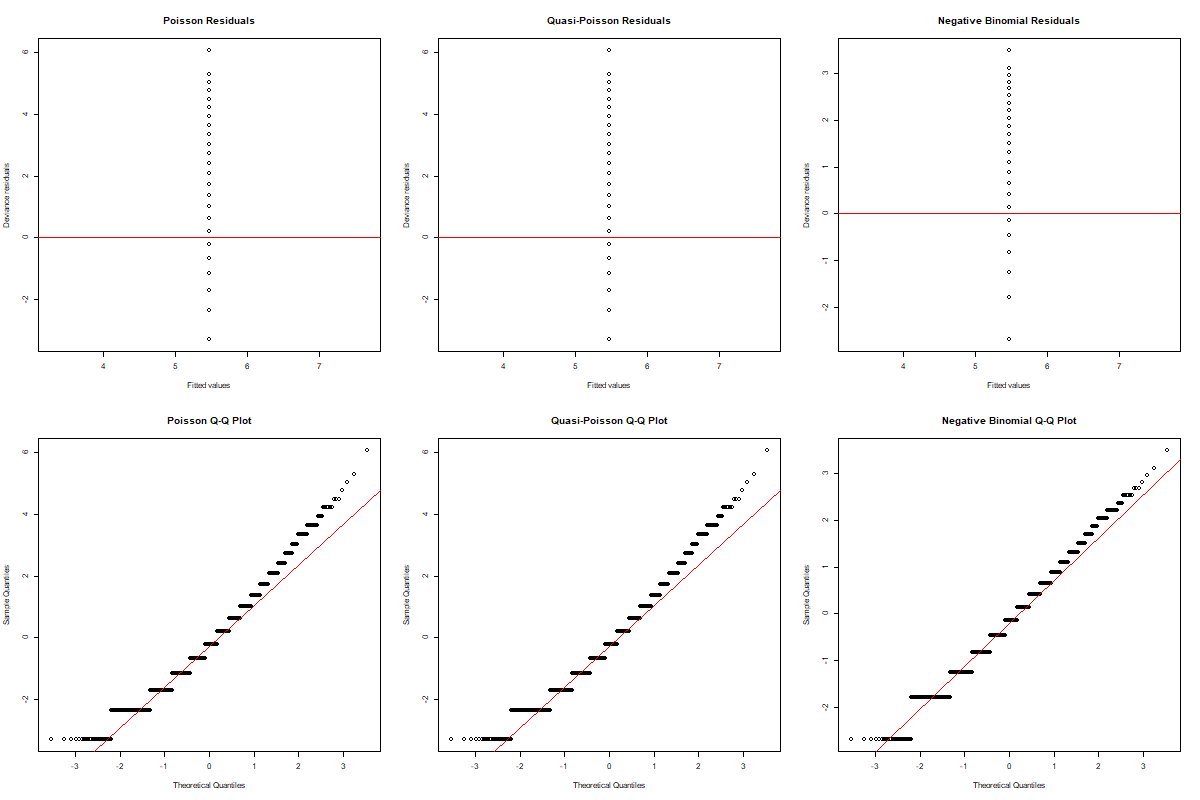

Supplement: S4 Fig — (TIF) [file pone.0318250.s005.tif]

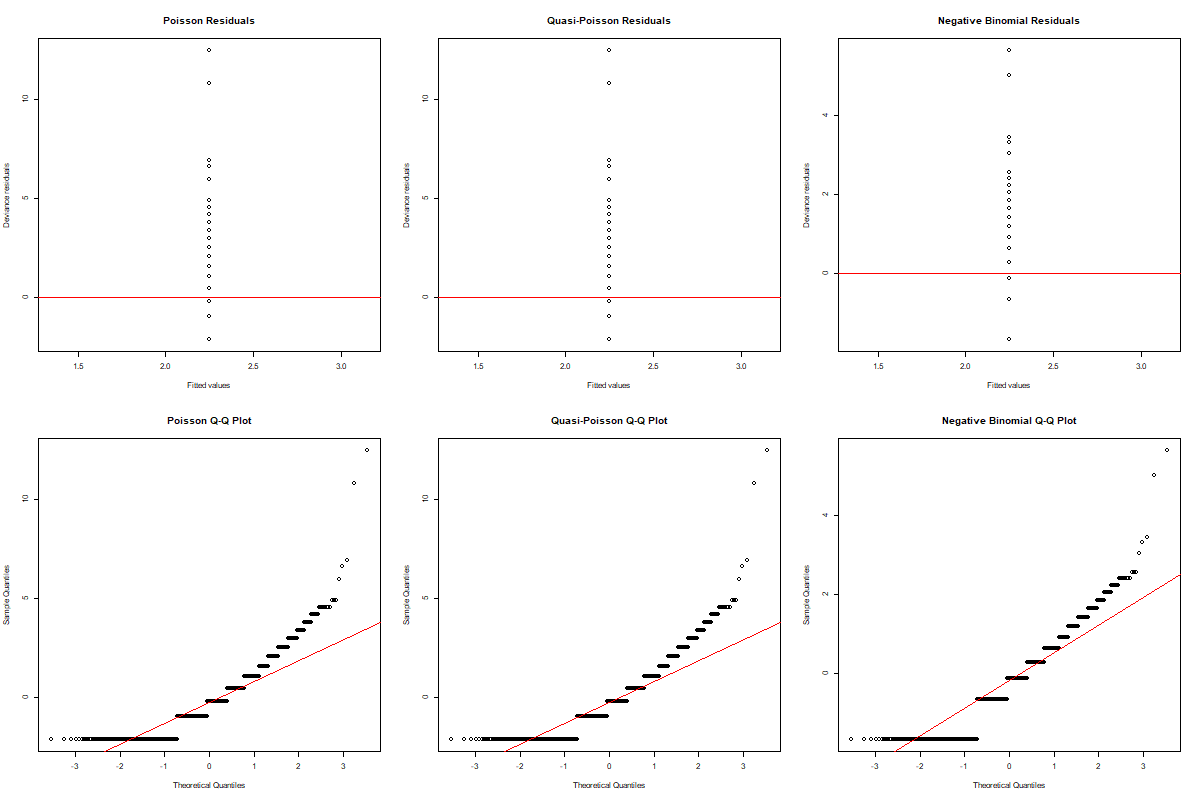

Supplement: S5 Fig — (TIF) [file pone.0318250.s006.tif]

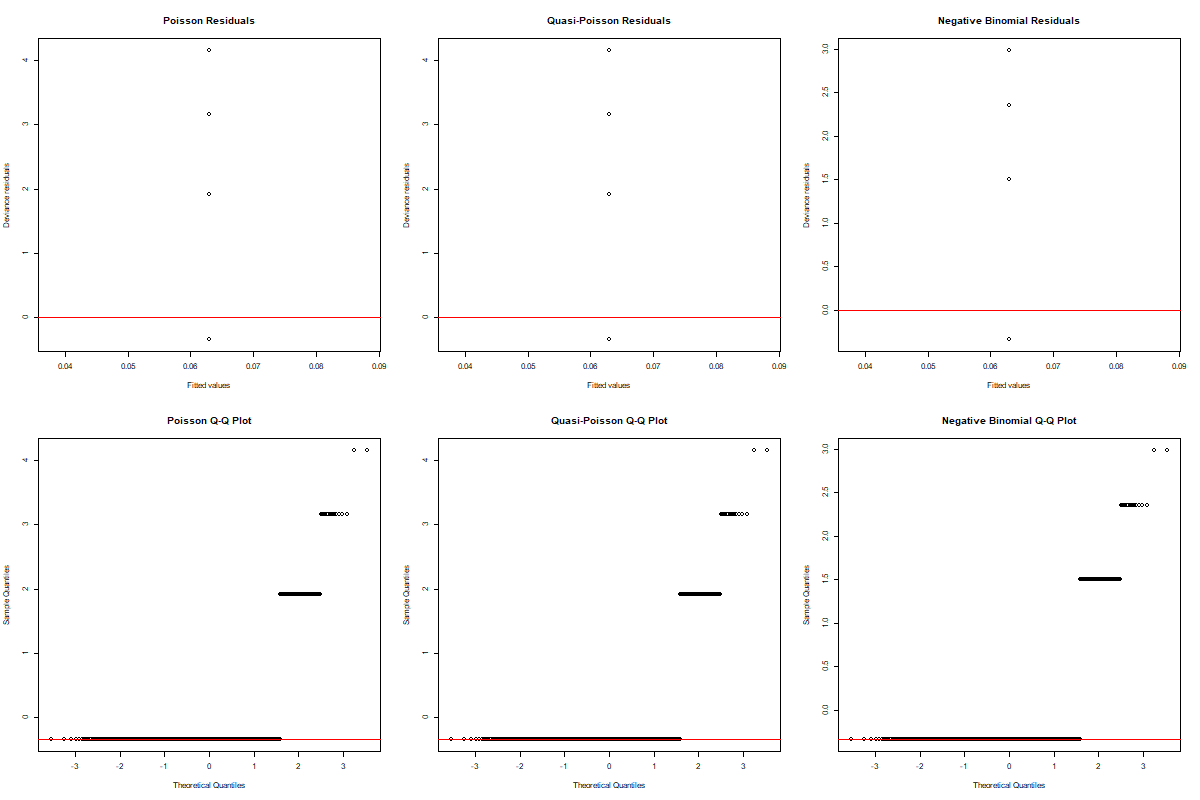

Supplement: S6 Fig — (TIF) [file pone.0318250.s007.tif]

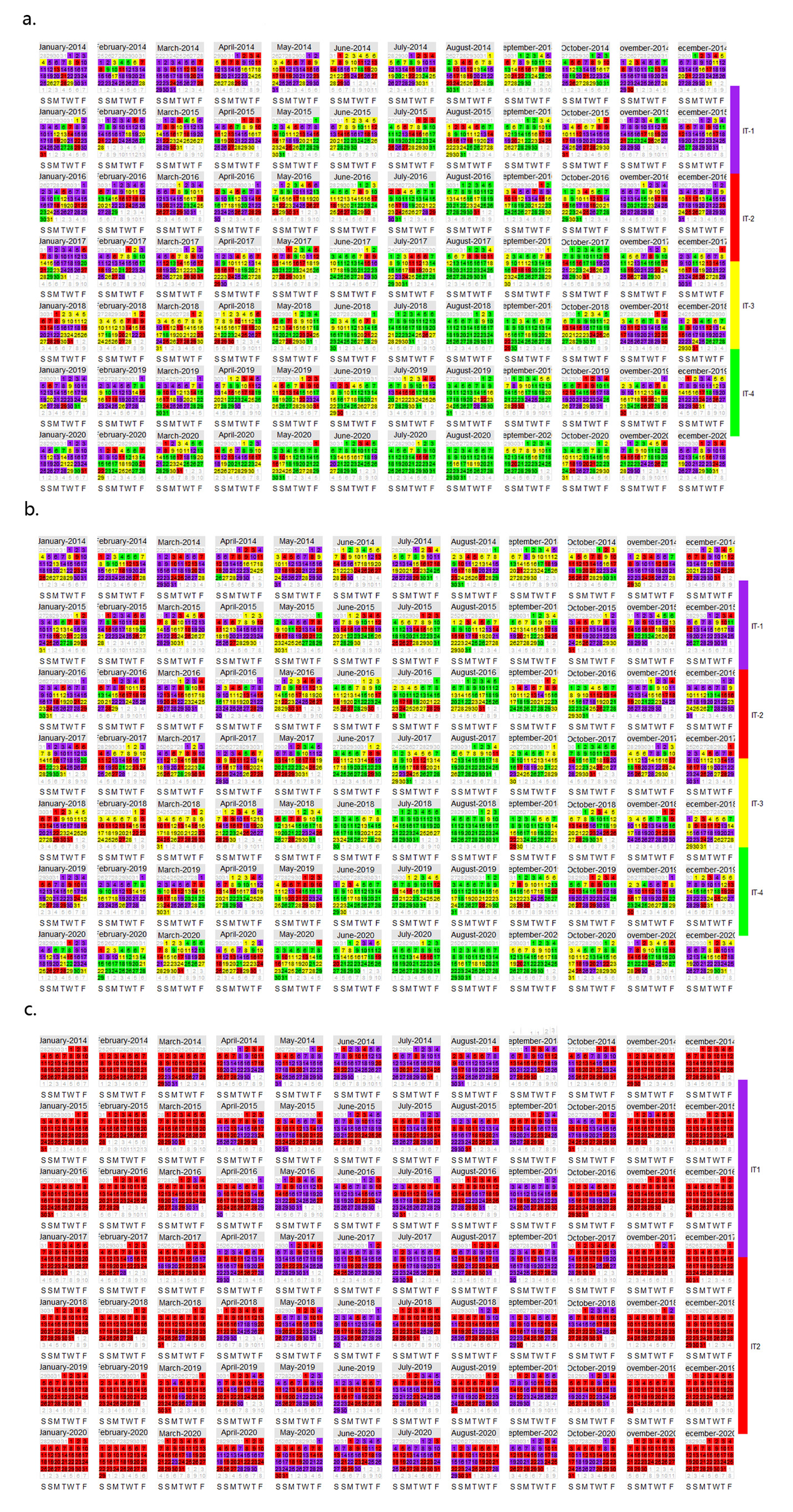

Supplement: S7 Fig — a, PM2.5; b, PM10; c, O3. (TIF) [file pone.0318250.s008.tif]

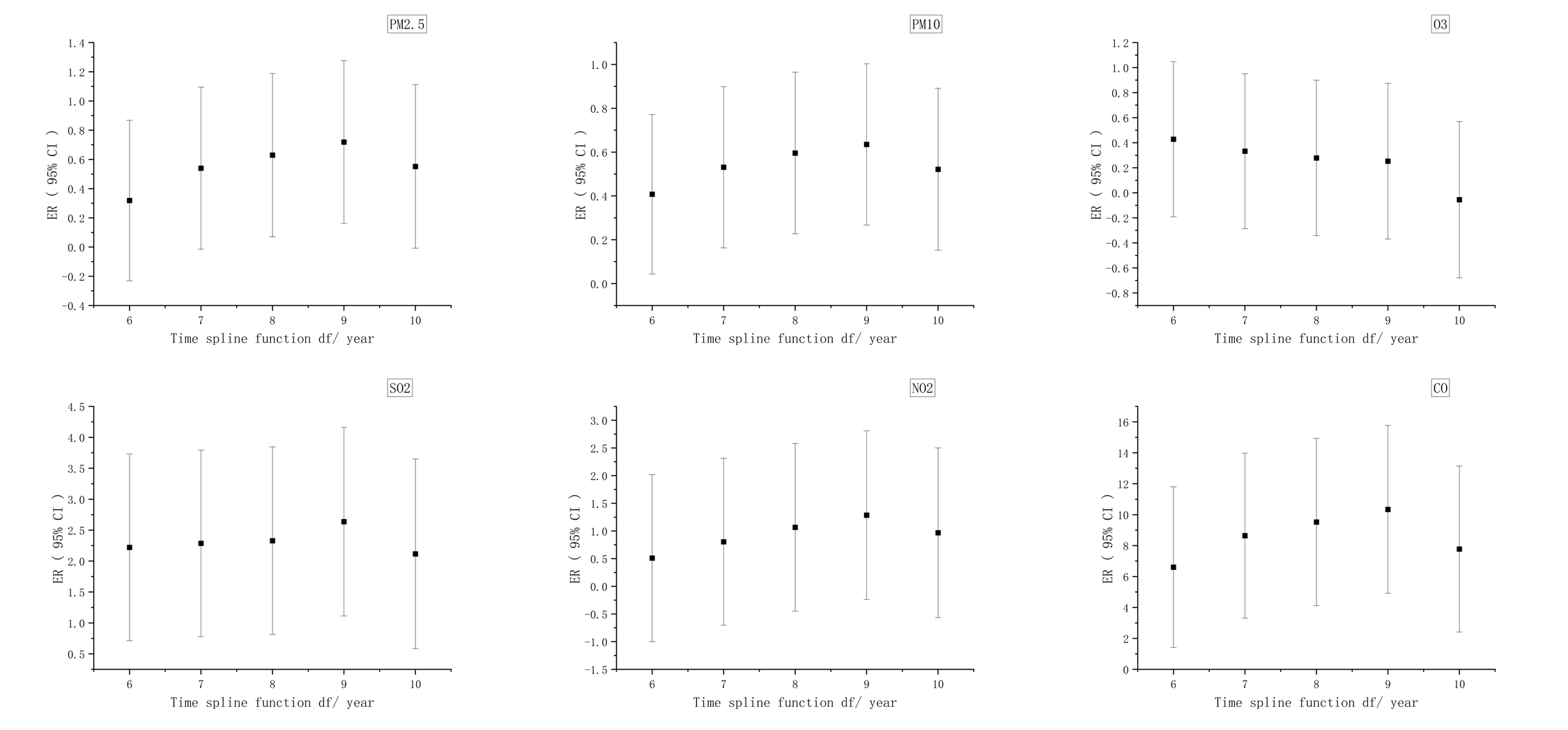

Supplement: S8 Fig — (TIF) [file pone.0318250.s009.tif]

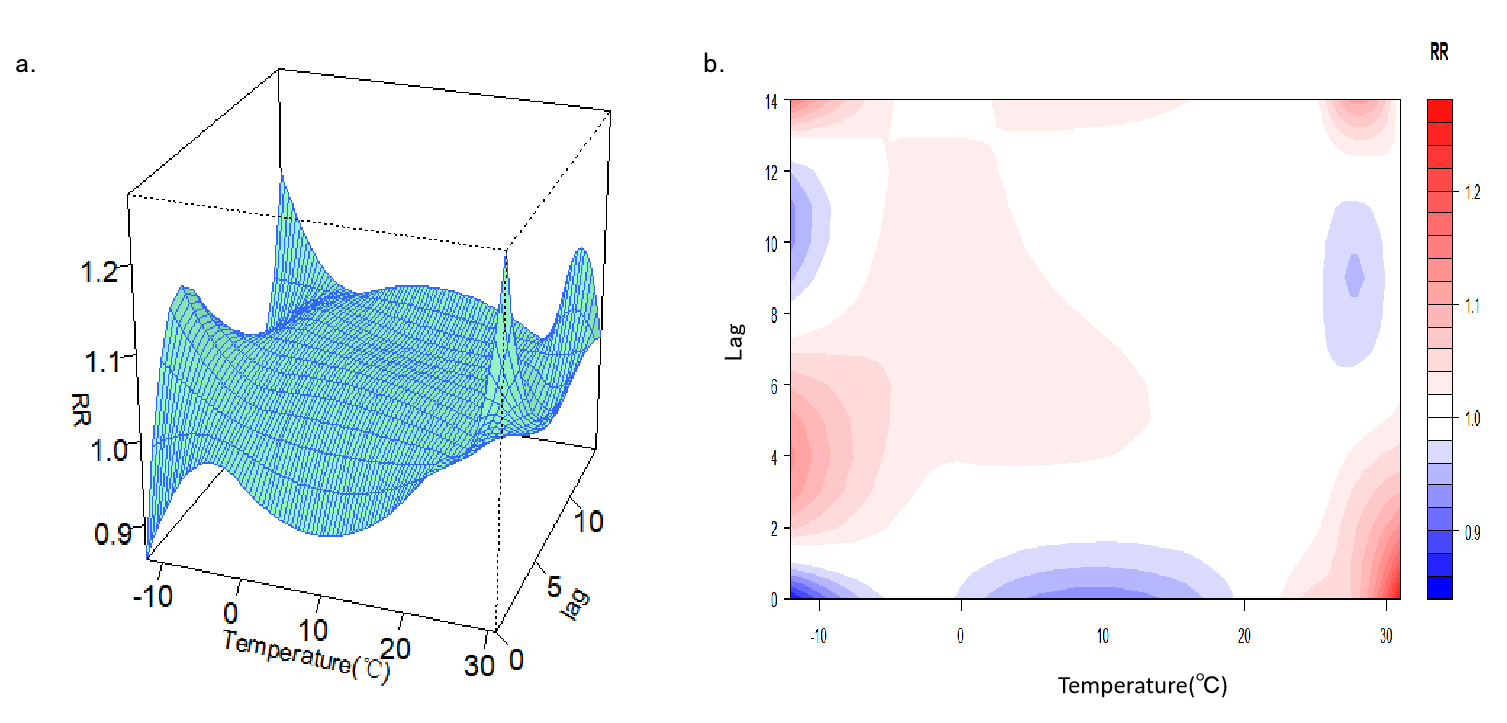

Supplement: S9 Fig — (TIF) [file pone.0318250.s010.tif]

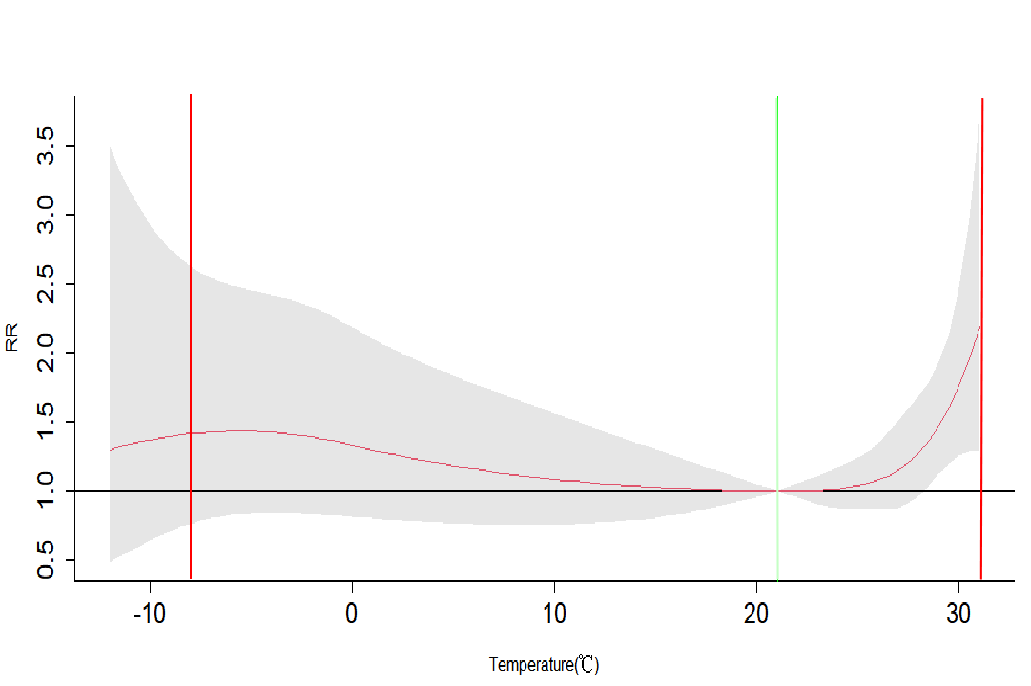

Supplement: S10 Fig — (TIF) [file pone.0318250.s011.tif]

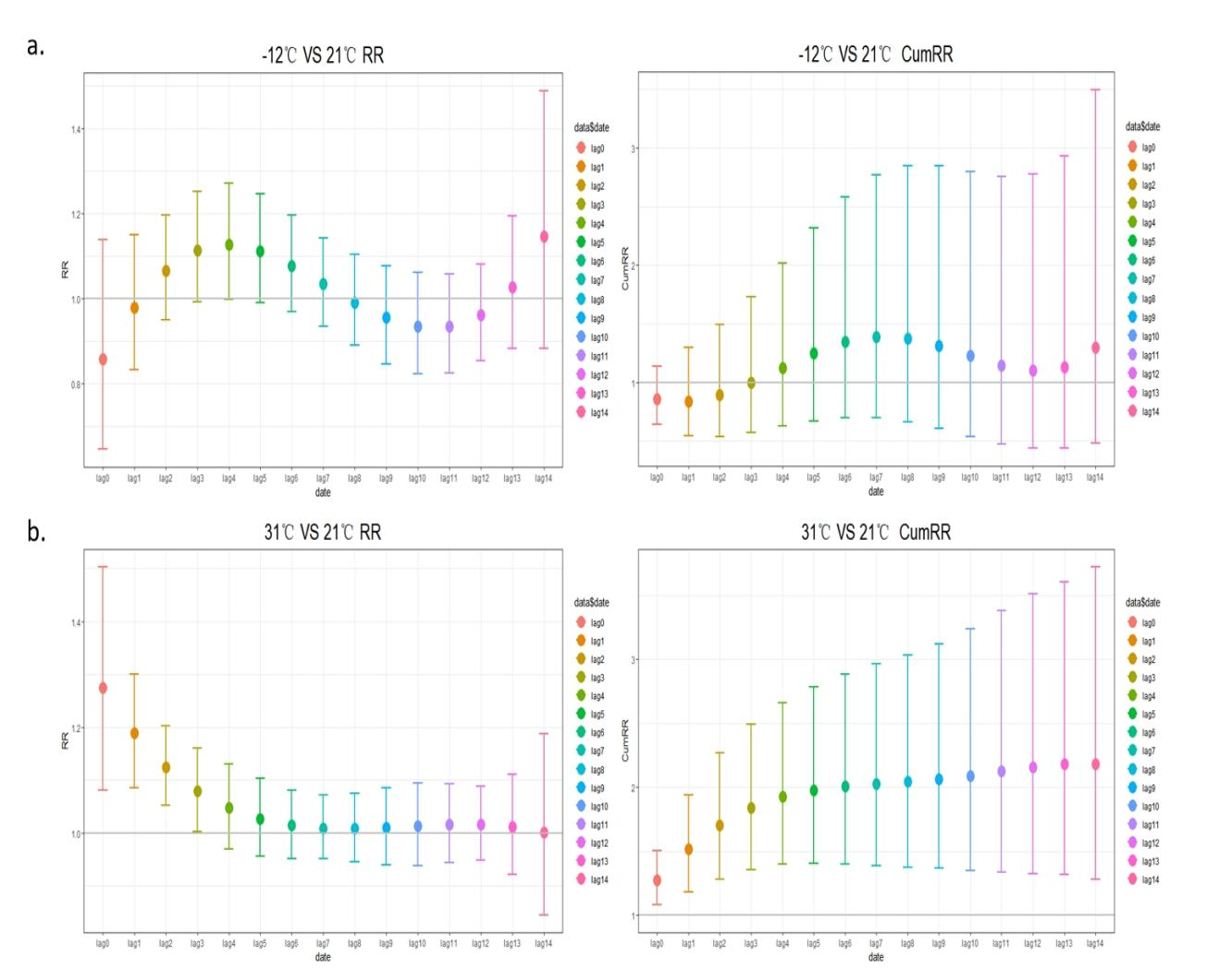

Supplement: S11 Fig — a, Minimum temperature(-12°C); b, Maximum temperature (31°C). (TIF) [file pone.0318250.s012.tif]

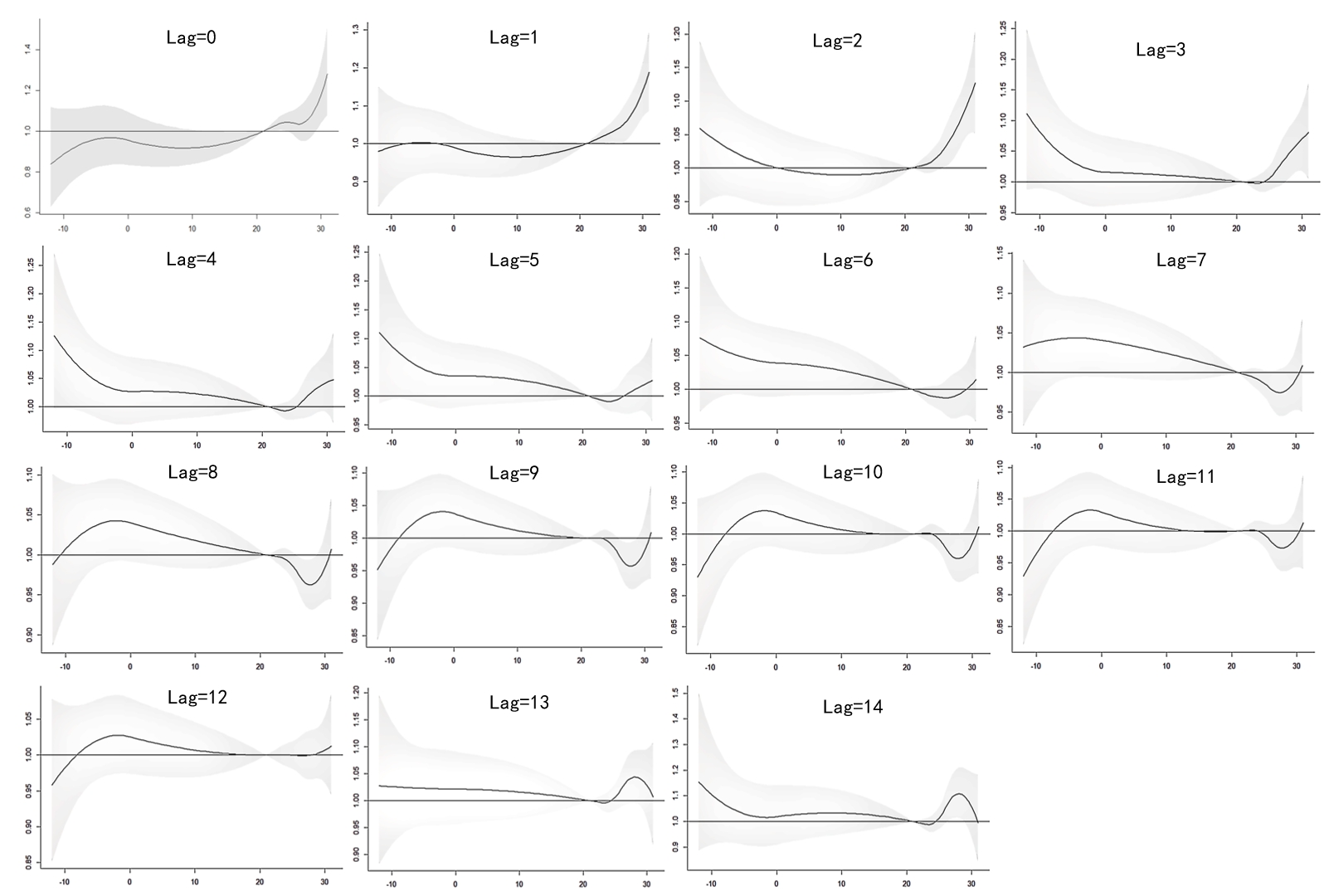

Supplement: S12 Fig — (TIF) [file pone.0318250.s013.tif]
